# Environmental Effects with Frozen-Density Embedding in Real-Time Time-Dependent Density Functional Theory Using Localized Basis Functions

**DOI:** 10.1021/acs.jctc.0c00603

**Published:** 2020-07-27

**Authors:** Matteo De Santis, Leonardo Belpassi, Christoph R. Jacob, André Severo Pereira Gomes, Francesco Tarantelli, Lucas Visscher, Loriano Storchi

**Affiliations:** † Dipartimento di Chimica, Biologia e Biotecnologie, Università degli Studi di Perugia, Via Elce di Sotto 8, 06123 Perugia, Italy; ‡ Istituto di Scienze e Tecnologie Chimiche (SCITEC), Consiglio Nazionale delle Ricerche c/o Dipartimento di Chimica, Biologia e Biotecnologie, Università degli Studi di Perugia, Via Elce di Sotto 8, 06123 Perugia, Italy; § Institute of Physical and Theoretical Chemistry, Technische Universität Braunschweig, Gaußstr. 17, 38106 Braunschweig, Germany; ∥ Univ. Lille, CNRS, UMR 8523-PhLAM-Physique des Lasers Atomes et Molécules, F-59000 Lille, France; ⊥ Theoretical Chemistry, Faculty of Science, Vrije Universiteit Amsterdam, De Boelelaan 1083, 1081 HV Amsterdam, The Netherlands; # Dipartimento di Farmacia, Università degli Studi ‘G. D’Annunzio’, Via dei Vestini 31, 66100 Chieti, Italy

## Abstract

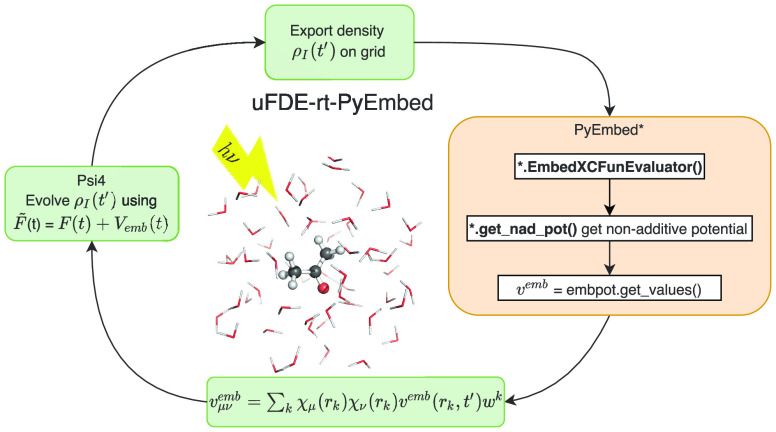

Frozen-density embedding (FDE) represents a versatile embedding scheme to describe the environmental effect on electron dynamics in molecular systems. The extension of the general theory of FDE to the real-time time-dependent Kohn–Sham method has previously been presented and implemented in plane waves and periodic boundary conditions [PavanelloM.; J. Chem. Phys.2015, 142, 1541162590387510.1063/1.4918276]. In the current paper, we extend our recent formulation of the real-time time-dependent Kohn–Sham method based on localized basis set functions and developed within the Psi4NumPy framework to the FDE scheme. The latter has been implemented in its “uncoupled” flavor (in which the time evolution is only carried out for the active subsystem, while the environment subsystems remain at their ground state), using and adapting the FDE implementation already available in the PyEmbed module of the scripting framework PyADF. The implementation was facilitated by the fact that both Psi4NumPy and PyADF, being native Python API, provided an ideal framework of development using the Python advantages in terms of code readability and reusability. We employed this new implementation to investigate the stability of the time-propagation procedure, which is based on an efficient predictor/corrector second-order midpoint Magnus propagator employing an exact diagonalization, in combination with the FDE scheme. We demonstrate that the inclusion of the FDE potential does not introduce any numerical instability in time propagation of the density matrix of the active subsystem, and in the limit of the weak external field, the numerical results for low-lying transition energies are consistent with those obtained using the reference FDE calculations based on the linear-response TDDFT. The method is found to give stable numerical results also in the presence of a strong external field inducing nonlinear effects. Preliminary results are reported for high harmonic generation (HHG) of a water molecule embedded in a small water cluster. The effect of the embedding potential is evident in the HHG spectrum reducing the number of the well-resolved high harmonics at high energy with respect to the free water. This is consistent with a shift toward lower ionization energy passing from an isolated water molecule to a small water cluster. The computational burden for the propagation step increases approximately linearly with the size of the surrounding frozen environment. Furthermore, we have also shown that the updating frequency of the embedding potential may be significantly reduced, much less than one per time step, without jeopardizing the accuracy of the transition energies.

## 1 Introduction

The last decade has seen a growing interest in the electron dynamics taking place in molecules subjected to an external electromagnetic field. Matter–radiation interaction is involved in many different phenomena ranging from weak-field processes, i.e., photoexcitation, absorption, scattering, light harvesting in dye-sensitized solar cells,^1,2^ and photoionization to strong-field processes encompassing high harmonic generation,^3,4^ optical rectification,^5,6^ multiphoton ionization,^7^ and the above threshold ionization.^8^ Furthermore, the emergence of new free electron laser (FEL) and attosecond methodologies^9,10^ opened an area of research in which experiments can probe electron dynamics and chemical reactions in real time and the movement of electrons in molecules may be controlled. These experiments can provide direct insights into bond breaking^11−13^/forming^14^ and ionization^15,16^ by directly probing nuclear and electron dynamics.

Real-time time-dependent electronic-structure theory, in which the equation of motion is directly solved in the time domain, is clearly the most promising for investigating time-dependent molecular response and electronic dynamics. The recent progress in the development of these methodologies is impressive (see, for instance, a recent review by Li et al.^17^). Among different approaches, because of its compromise between accuracy and efficiency, the real-time time-dependent density functional theory (rt-TDDFT) is becoming very popular. The main obstacle to implementing the rt-TDDFT method involves the algorithmic design of a numerically stable and computationally efficient time-evolution propagator. This typically requires the repeated evaluation of the effective Hamiltonian matrix representation (Kohn–Sham matrix) at each time step. Despite the difficulties in realizing a stable time propagator scheme, there are very appealing features in a real-time approach to TDDFT, such as the absence of explicit exchange-correlation kernel derivatives^18^ or divergence problems appearing in the response theory, and one has the possibility of obtaining all frequency excitations at the same cost. Furthermore, the method is suitable to treat complex nonlinear phenomena and external fields with an explicit shape, which is a key ingredient in the quantum optimal control theory.^19^

Several implementations have been presented^20−22^ after the pioneering work of Theilhaber^23^ and Yabana and Bertsch.^24^ Many of them rely on the real-space grid methodology,^24^ with Siesta and Octopus being the most recent ones.^25,26^ Alternative approaches employ plane waves such as in Qbox^27^ or QUANTUM ESPRESSO,^28,29^ and analytic atom-centered Gaussian basis implementations (i.e., Gaussian,^30,31^ NWChem,^32^ Q-Chem^33,34^) also have gained popularity. The scheme has been also extended to include relativistic effects at the highest level. Repisky et al. proposed the first application and implementation of relativistic TDDFT to atomic and molecular systems^35^ based on the four-component Dirac Hamiltonian, and almost simultaneously Goings et al.^36^ published the development of X2C Hamiltonian-based electron dynamics and its application to the evaluation of UV–vis spectra. Very recently, some of us presented an rt-TDDFT implementation^37,38^ based on state-of-the-art software engineering approaches (i.e., including interlanguage communication between high-level languages such as Python, C, FORTRAN, and prototyping techniques). The method, based on the design of an efficient propagation scheme within the Psi4NumPy^39^ framework, was also extended to the relativistic four-component framework based on the BERTHA code^40−42^ (more specifically based on the recently developed PyBERTHA,^37,38,43,44^ i.e., the Python API of BERTHA).

The applications of the rt-TDDFT approach encompass studies of linear^45^ and nonlinear optical response properties,^25,46^ molecular conductance,^47^ singlet–triplet transitions,^48^ plasmonic resonances magnetic circular dichroism,^49^ core excitation, photoinduced electric current, spin-magnetization dynamics,^50^ and Ehrenfest dynamics.^51,52^ Moreover, there have been many studies in the relativistic and quasi-relativistic framework, ranging from X-ray near-edge absorption^53^ to nonlinear optical properties^54^ to chiroptical spectroscopy.^55^

Mostly, initial applications of the real-time methodology to chemical systems were largely focused on the electron dynamics and optical properties of isolated target systems. However, it is widely recognized that these phenomena are extremely sensitive to the polarization induced by the environment, such that the simulation on an isolated molecule is usually not sufficient even for a qualitative description. A number of studies aiming at including the effect of a chemical environment within rt-TDDFT have appeared in the literature. They are based on the coupling of rt-TDDFT with the QM/MM approach, which includes the molecular environment explicitly and at a reduced cost using classical mechanical description^31,56^ or in a polarizable continuous medium (PCM), where the solvent degrees of freedom are replaced by an effective classical dielectric.^57−59^ One of the challenges, however, in the dynamical description of the environment is that the response of the solvent is not instantaneous; thus, these approaches have been extended to include the nonequilibrium solvent response.^60−63^ A recent extension also considers nonequilibrium cavity field polarization effects for molecules embedded in a homogeneous dielectric.^64^

Going beyond a classical description for the environment, very recently, Koh et al.^65^ combined the rt-TDDFT method with the block-orthogonalized Manby–Miller theory^66^ to accelerate the rt-TDDFT simulations; the approach is also suitable for cheaply accounting for the solvation effect on the molecular response. Another fully quantum mechanical approach to include environmental effects in the molecular response property is based on the frozen-density embedding (FDE) scheme.^67−69^ FDE is a DFT-in-DFT embedding method that allows one to partition a larger Kohn–Sham system into a set of smaller, coupled Kohn–Sham subsystems. In addition to the computational advantage, FDE provides physical insights into the properties of embedded systems and the coupling interactions between them.^70^

For electronic ground states, the theory and methodology were introduced by Wesołowski and Warshel,^71^ based on the approach originally proposed by Senatore and Subbaswamy,^72^ and later Cortona,^73^ for solid-state calculations. It has been further generalized^74,75^ and directed to the simultaneous optimization of the subsystem electronic densities. Within the linear-response formalism, Casida and Wesołowski put forward a formal TDDFT generalization^76^ of the FDE scheme. Neugebauer^77,78^ then introduced coupled FDE, a subsystem TDDFT formulation that removed some of the approximations made in the initial TDDFT-FDE implementations. Recently, the approach has been further extended^79,80^ to account for charge-transfer excitations, taking advantage of an exact FDE scheme.^81−85^

A DFT subsystem formulation of the real-time methodology has been presented in a seminal work by Pavanello and co-workers^70^ together with its formulation within the FDE framework. They showed that the extension of FDE to rt-TDDFT can be done straightforwardly by updating the embedding potential between the systems at every time step and evolving the Kohn–Sham subsystems in time simultaneously. Its actual implementation, based on the use of plane waves and ultrasoft pseudopotentials,^29,70^ showed that the updating of the embedding potentials during the time evolution of the electron density does not affect the numerical stability of the propagator. The approach may be approximated and devised in the so-called “uncoupled” scheme where the density response to the external field is limited to one active subsystem while keeping the densities of the other subsystems frozen in time. Note that also in this uncoupled version the embedding potential is time-dependent and needs to be recomputed and updated during the time propagation. However, the propagation scheme is restricted to the active subsystem and the approach is promising to include environmental effects in real-time simulation. Numerous applications within the context of the linear-response TDDFT showed that an uncoupled FDE is sufficient for reproducing supermolecular results with good accuracy even in the presence of hydrogen bonds as long as there are no couplings in the excitations between the systems.

In this work, we extend rt-TDDFT based on localized basis functions to the FDE scheme in its uncoupled version (uFDE-rt-TDDFT), taking advantage of modern software engineering and code reusability offered by the Python programming language. We devised a unified framework based on Python in which the high interoperability allowed the concerted and efficient use of the recent rt-TDDFT procedure, which some of us have implemented in the framework of the Psi4Numpy API^37,38^ and the PyADF API.^86^ The rt-TDDFT procedure has served as the main interface where the PyADF methods, which gave direct access to the key quantities necessary to devise the FDE scheme, can be accessed within a unified framework. Since in this work we introduced a new flavor of the rt-TDDFT Psi4Numpy-based program, to avoid confusions, henceforth in this article, we will refer to the aforementioned rt-TDDFT based on Psi4Numpy as Psi4-rt, while its extension to the FDE subsystem framework will be referred to as Psi4-rt-PyEmbed.

In Section 2, we review the fundamentals of FDE and its extension to the rt-TDDFT methodology. In Section 3, computational details are given with a specific focus on the interoperability of the various codes we merged and used: Psi4Numpy,^39^ XCFun,^87^ and PyADF,^86^ including the PyEmbed module recently developed by some of us. In Section 4, we report and comment on the results of the calculations we performed on excitation transitions for different molecular systems, including a water–ammonia complex, a water cluster, and a more extend acetone-in-water cluster case. Finally, we give some preliminary results about the applicability and numerical stability of the method in the presence of an intense external field inducing strong nonlinear effects such as high harmonic generation (HHG) in the active system. Concluding remarks and perspectives are finally given in Section 5.

## 2 Theory

In this section, we briefly review the theoretical foundations of the FDE scheme and its extension to the rt-TDDFT methodology. As mentioned above, a previous implementation was presented by Pavanello et al.^70^ using plane waves and ultrasoft pseudopotentials. We refer the interested reader to this seminal work for a general theoretical background and for additional details of the FDE-rt-TDDFT formal derivation.

### 2.1 Subsystem DFT and Frozen-Density Embedding Formulation

In the subsystem formulation of DFT, the entire system is partitioned into *N* subsystems, and the total density ρ_tot_(***r***) is represented as the sum of electron densities of the various subsystems [i.e., ρ_*a*_(***r***) (*a* = 1, ..., *N*)]. Focusing on a single subsystem, we can consider the total density as partitioned in only two contributions as
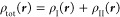
1The total energy of the system can then be written as
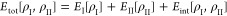
2with the energy of each subsystem (*E*_*i*_[ρ_*i*_], with *i* = I, II) given according to the usual definition in DFT as
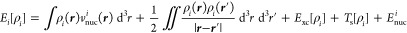
3In the above expression, *v*_nuc_^*i*^(***r***) is the nuclear potential due to the set of atoms that defines the subsystem and *E*_nuc_^*i*^ is the related nuclear repulsion energy. *T*_s_[ρ_*i*_] is the kinetic energy of the auxiliary noninteracting system, which is, within the Kohn–Sham (KS) approach, commonly evaluated using the KS orbitals. The interaction energy is given by the expression
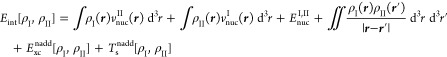
4with *v*_nuc_^I^ and *v*_nuc_^II^ being the nuclear potentials due to the set of atoms associated with subsystems I and II, respectively. The repulsion energy for nuclei belonging to different subsystems is described by the *E*_nuc_^I,II^ term. The nonadditive contributions are defined as
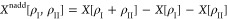
5with *X* = *E*_xc_, *T*_s_. These terms arise because both exchange correlation and kinetic energy, in contrast to the Coulomb interaction, are not linear functionals of the density.

The electron density of a given fragment (ρ_I_ or ρ_II_ in this case) can be determined by minimizing the total energy functional (eq 2) with respect to the density of the fragment while keeping the density of the other subsystem frozen. This procedure is the essence of the FDE scheme and leads to a set of Kohn–Sham-like equations (one for each subsystem)
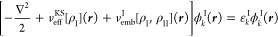
6which are coupled by the embedding potential term *v*_emb_^I^(***r***), which carries all dependence on the other fragment’s density. In this equation, *v*_eff_^KS^[ρ_I_](***r***) is the KS potential calculated on the basis of the density of subsystem I only, whereas the embedding potential takes into account the effect of the other subsystem (which we consider here as the complete environment). In the framework of the FDE theory, *v*_emb_^I^(***r***) is explicitly given by
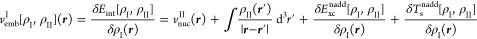
7where the nonadditive exchange correlation and kinetic energy contributions are defined as the difference between the associated exchange-correlation and kinetic potentials defined using ρ_tot_(***r***) and ρ_I_(***r***). For both potentials, one needs to account for the fact that only the density is known for the total system so that potentials that require input in the form of KS orbitals are prohibited. For the exchange-correlation potential, one may make use of accurate density functional approximations, and its quality is therefore similar to that of ordinary KS. The potential for the nonadditive kinetic term (, in eq 7) is more problematic as less accurate orbital-free kinetic energy density functionals (KEDFs) are available for this purpose. Examples of popular functional approximations applied in this context are the Thomas–Fermi (TF) kinetic energy functional^88^ or the GGA functional PW91k.^89^ These functionals have been shown to be accurate in the case of weakly interacting systems including hydrogen-bond systems, whereas their use in subsystems interacting with a larger covalent character is problematic (see ref (81) and references therein). The research for more accurate KEDFs is a key aspect for the applicability of the FDE scheme as a general scheme, including the partitioning of the system also breaking covalent bonds.^90^

In general, the set of coupled equations that arises in the FDE scheme for the subsystems has to be solved iteratively. Typically, one may employ a procedure of “freeze-and-thaw”, where the electron density of the active subsystem is determined keeping frozen the electron density of the other subsystems, which is then frozen when the electron density of the other subsystems is worked out. This procedure may be repeated many times until all subsystems’ densities are converged. In this case, the FDE scheme can be seen as an alternative formulation of the conventional KS-DFT approach for large systems (by construction, it scales linearly with the number of subsystems). The update of the density for (part of) the environment can be important when trial densities obtained from isolated subsystems are not a very good starting point, as is the case for ionic species.^91−93^

The implementation of FDE is relatively straightforward, in that the *v*_emb_^I^(***r***) potential is a one-electron operator that needs to be added to the usual KS Hamiltonian. When using localized basis functions, the matrix representation of the embedding potential (**V**^emb^) may be evaluated using numerical integration grids similar to those used for the exchange-correlation term in the KS method. This contribution is then added to the KS matrix, and the eigenvalue problem is solved in the usual self-consistent field manner.

We note that, irrespective of whether one or many subsystem densities are optimized, the matrix **V**^emb^ needs to be updated during the SCF procedure because it also depends on the density of the active subsystem (see eq 7).

Going beyond the ground state is necessary to access many interesting properties, which for DFT are expressed via the response theory,^76−78,94,95^ such as electronic absorption^96^ or NMR shielding,^93,97^ and for which FDE has been shown to work properly since these are quite often relatively local. In a response formulation, the embedding potential as well as its derivatives enters the equations and, if more than one subsystem is allowed to react to the external perturbations,^77,78,94,95^ the derivatives of the embedding potential introduce the coupling in the subsystems’ response (as the embedding potential introduces the coupling of the subsystems’ electronic structure in the ground state).

While such couplings in response may be very important in certain situations, such as for strongly interacting systems^79,80^ or for extensive properties,^78^ disregarding them can still provide a very accurate picture, notably for localized excited states.^91,96^ In this simplified “uncoupled” framework, one considers only the response of the subsystem of interest (and thus the embedding potential and its derivative with respect to this subsystem’s density). While neglecting environment response may seem like a drastic approximation, good performance relative to supermolecular reference data has been obtained for excitation energies of a chromophore in a solvent or a crystal environment, even when only retaining the embedding potential.^91^ We will therefore employ this framework in the following.

### 2.2 Real-Time Time-Dependent Kohn–Sham Method and Its Extension to FDE

The time-dependent equation for the Kohn–Sham method can be conveniently formulated in terms of the Liouville–von Neumann (LvN) equation. In an orthonormal basis set, the LvN equation reads
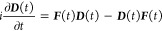
8where *i* is the imaginary unit and ***D***(*t*) and ***F***(*t*) are the one-electron density matrix and time-dependent Kohn–Sham matrix, respectively. The above equation holds in both the nonrelativistic and relativistic four-component formulations.^35,40^

In a nonrelativistic framework, the Kohn–Sham matrix (***F***(*t*)) is defined as
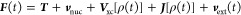
9where ***T*** and ***v***_nuc_ are the one-electron nonrelativistic kinetic energy and intramolecular nuclear attraction terms, respectively. The explicit time dependence of ***F***(*t*) is due to the time-dependent external potential ***v***_ext_(*t*), which accounts for the interaction of the molecular system with an applied external electric field. Even in the absence of an external field, the Fock operator is implicitly dependent on time through the density matrix ***D***(*t*) in the Coulomb (***J***[ρ(*t*)]) and exchange-correlation terms (***V***_xc_[ρ(*t*)]).

The propagation in time of the density matrix can be expressed as
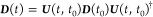
10where ***U***(*t*,*t*_0_) is the matrix representation of the time-evolution operator.

If we start (initial condition, i.e., initial time *t*_0_) with the electronic ground-state density matrix and use as orthonormal basis the ground-state molecular orbitals, ***D***(*t*_0_) assumes the form
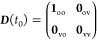
where **1**_oo_ is the identity matrix over the occupied orbital space of size *n*_occ_ (total number of electrons). The ***D*** matrix has the dimension of *n*_tot_ (*n*_tot_ = *n*_occ_ + *n*_virt_), which is the total number of basis functions.

In our implementation, which uses a basis set of atomic centered (AO) Gaussian-type functions, the ground-state molecular orbitals are conveniently used as the reference orthonormal basis and at time *t* the Fock and density matrices are related to their AO basis representation simply by
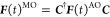
11where the ***C*** matrix contains the reference MO expansion coefficients. The same coefficients satisfy a similar relation for ***D***(*t*)^MO^
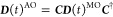
12In a finite time interval, the solution of the Liouville–von Neumann equation consists of the calculation of the Fock matrix at discrete time steps and in propagating the density matrix in time.

In the most general case, where the Fock operator depends on time even in the absence of external fields, the time-evolution operator can be expressed by means of a Dyson-like series
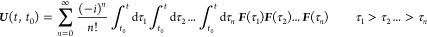
13which in compact notation, using the time-ordering operator , reads as
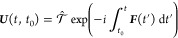
14The time ordering is necessary since ***F***(*t*) at different times does not necessarily commute ([***F***(*t*),***F***(*t*′)] ≠ 0). Typically, this time-ordering problem is overcome by exploiting the composition property of the time-evolution operator (***U***(*t*,*t*_0_) = ***U***(*t*,*t*_1_) ***U***(*t*_1_,*t*_0_)) and discretizing the time using a small time step. It is clear that the exact time ordering can be achieved only in the limit of an infinitesimal time step. Many different propagation schemes have been proposed^98^ in the context of rt-TDDFT. Among others, we mention the Crank–Nicholson,^99^ Runge–Kutta,^100^ or Magnus^101,102^ methods.

The Magnus expansion has found the widest application, in particular, in those implementations that employ localized basis set functions for which matrix exponentiation can be performed exactly via matrix diagonalization. Typically, the Magnus expansion is truncated to the first order evaluating the integral over time using numerical quadrature, provided that the time interval Δ*t* is sufficiently short. Using the midpoint rule, the propagator becomes
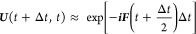
15This approach, also referred to as the second-order midpoint Magnus propagator, is unitary by construction, provided that ***F*** is Hermitian. This scheme exhibits an error that is proportional to (Δ*t*)^3^. The expression in eq 15 coincides with the so-called modified-midpoint unitary transform time-propagation scheme originally introduced by Schlegel et al.^21^

The ***F*** matrix at time *t* + Δ*t*/2, where no density is available, can be obtained using an iterative series of extrapolations and interpolations at each time. Note that if this predictor/corrector procedure is converged in a self-consistent manner the second-order midpoint Magnus propagator preserves the time reversal symmetry, which is an exact property of the equation of motion in the absence of a magnetic field. The predictor/corrector scheme is a key factor in preserving the numerical stability of the propagation with a range of algorithms that can be applied in this context.^103^ We have recently implemented a particularly stable predictor/corrector scheme, originally proposed by Repisky et al.,^35^ in the interactive quantum chemistry programming environment Psi4NumPy.^37−39^

The methodology that we have described above can be straightforwardly extended to the subsystem density functional theory framework and in particular to FDE (FDE-rt-TDDFT).^70^ In the present work, we consider one active subsystem and keep frozen the density of the environment along the time propagation (uncoupled scheme, which we will refer to as uFDE-rt-TDDFT). Thus, an LvN-type equation is solved in the space of the active subsystem. The only modification to eq 8 is in the definition of the effective Hamiltonian matrix representation, which now refers to the active subsystem (***F***^I^(*t*) = ***T***^I^ + ***v***_nuc_^I^ + ***V***_xc_[ρ^I^(*t*)] + ***J***[ρ^I^(*t*)] + ***v***_ext_(*t*)) and to which the matrix representation of the embedding potential (***V***^emb^(*t*)) is added to take into account the effect of the environment. The propagation scheme itself remains unaltered.

As in the case for the ground state, in which the change of the active subsystem density requires that ***V***^emb^ is updated at each SCF iteration, the time propagation of the electron density will introduce a time dependence in ***V***^emb^ even though the environment densities are kept frozen at their ground-state value (due to the use of the uncoupled scheme).

Thus, the ***V***^emb^ matrix needs to be updated during the propagation. In the present implementation, we use atomic centered Gaussian function as the basis set for the active subsystem and evaluate the *V*_μν_^emb^ matrix elements numerically.^91^ We will show that the numerical noise associated with the construction of the embedding potential introduced by this scheme does not affect the numerical stability of the density matrix propagation in the linear and nonlinear regimes. In the following sections, we will also demonstrate, for a specific application, that the updating frequency of the embedding potential may be significantly reduced (much less than one per time step used to solve the LvN equation) without jeopardizing the accuracy.

As usual, the key quantity in a real time simulation is the time-dependent electric dipole moment μ⃗(*t*). Each Cartesian component *p* (with *p* = *x*, *y*, *z*) is given by
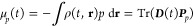
16where ***P***_*p*_ is the matrix representation of the *p*-th component of the electric dipole moment operator (see also eq 16). Since, in our uFDE-rt-TDDFT implementation, the time dependency response of the external field is due only from the active system, in the above expression (eq 16), all quantities refer to the active subsystem. The vector μ⃗(*t*) defines the polarization response to all orders and is easily computed by the electronic density at any time, *t*. From this quantity, one can then compute both linear and nonlinear properties.

In the linear-response regime, each component of the electric dipole moment, μ_*p*_(ω), with an external field *E*_*q*_ in the direction *q* (with *q* = *x*, *y*, *z*), is given in the frequency space by
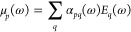
17The components depend on the polarizability tensor (α_*pq*_) through the Fourier transformation of the *q*-component of the applied field. The dipole strength function *S*(ω) is related to the imaginary part of the frequency-dependent linear polarizability by
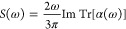
18In our implementation,^37^ the perturbation can be chosen to be either an impulsive kick or a continuous wave whose amplitude is modulated by an analytic envelope function. Different explicit functional forms are available.^37,38^ In the case of an impulsive perturbation (***E***(*t*) = *k*δ(*t*) ***n***, where ***n*** is a unit vector representing the orientation of the field), we adopt the δ-analytic representation as proposed in ref (35). One of the best-known examples of nonlinear optical phenomena is HHG in atoms and molecules. HHG occurs via photoemission by the molecular system in a strong field and can also be computed from μ⃗(*t*).^104^ In this work, we calculate the HHG power spectrum for a particular polarization direction as the Fourier transform of the laser-driven induced dipole moment
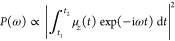
19Other suitable approaches have been investigated in the literature,^104^ but in all cases, the key quantity is μ⃗(*t*).

## 3 Computational Details and Implementation

In this section, we outline the computational strategy we adopted to implement the uFDE-rt-TDDFT scheme. We devised a multiscale approach where we take advantage of the real-time TDDFT reference procedure, recently implemented within the Psi4Numpy framework (i.e., the Psi4-RT program),^37,38^ while the FDE computational core relies on PyADF^86,105^ and makes use of its PyEmbed module, which some of us have recently developed.^106,107^ PyEmbed provides a Python implementation for computing the interaction energy (eq 4) and embedding potential (eq 7) from FDE on user-defined integration grids while using the XCFun library^18,87^ to evaluate nonadditive exchange-correlation and kinetic energy contributions. With PyEmbed, quantum chemistry codes require only minimal changes: functionality to provide electron densities and its derivatives, as well as the electrostatic potential, over the grid, as well as to read in the embedding potential, and add it as a one-electron operator in the Fock matrix.^91^ The PyADF scripting framework provides all of the necessary tools to manage various computational tasks and manipulate the relevant quantities for electronic-structure methods. The resulting Python code, referred to as Psi4-rt-PyEmbed, is available under GPLv3 license in ref (108). A data-set collection of computational results, including numerical data, parameters, and job input instructions used to obtain the absorption spectra of Sections 4.2, 4.3, and 4.5, is available in the Zenodo repository and can be freely accessed in ref (109).

### 3.1 Rapid Prototyping and Implementation

Psi4Numpy^39,110^ and PyADF^86,105^ both provide a Python interface, which greatly simplifies the computational workflow from input data to the results. PyADF is a quantum chemistry scripting framework that provides mechanisms for both controlling the execution of different computational tasks and for managing the communication between these tasks using Python object-oriented programming techniques. As we already mentioned, its built-in classes permit one to handle different aspects involved in the workflow as a single unit. All of the advantages coming from object-oriented programming (i.e., extensibility and inheritance) are readily available and allow us to incorporate a third-party scientific code and directly manipulate quantities coming from different codes (Psi4Numpy) in our case.

The Python HLL (high-level language), among others, permits one to formally express complex algorithms in comparatively few lines of codes. This makes it rather straightforward to let PyADF interact with Psi4Numpy native Python API. For the sake of completeness, we want to finally mention that, to accomplish our goal, we first had to port some of the frameworks (specifically XCFun, PyADF, and PyEmbed) to the new Python 3.0 standard (i.e., we forked the original code of the cited packages, and we made them publicly available at refs (111, 112)).

As an explicit example of the interoperativity achieved between different codes, we report in Algorithm 1 some basic directives used to compute those key quantities necessary for our uFDE-rt-TDDFT. The electron density of an active system is obtained via Psi4Numpy, while the electron density, the Coulomb potential, and nonadditive terms of the environment are managed using PyADF. These quantities can be easily mapped on a common numerical grid and used in PyEmbed to evaluate the relative nonadditive embedding potential. Thus, the geometry and basis set of the active system (in this specific case, a H_2_O molecule) are parsed at Line 7 and the ground-state wave-function object is returned by the *psi4.energy()* method. The corresponding electron density matrix is then obtained as a NumPy array by the *h2o*_*wfn* object. The electron density is mapped into a real-space grid representation using a preset numerical grid and used to populate a suitable object container (Lines 14–20). A ground-state calculation of the environment molecule (i.e., an NH_3_ molecule in this example) is carried out using the PyADF *run()* method (Line 23). In this case, we use the *adfsinglepointjob* method to execute the corresponding ADF calculation.^113^ We mention here that PyADF, despite its name, is not specific to this program but works with a number of different quantum chemistry codes. The density and Coulomb potential resulting from this calculation, which are represented on a common numerical grid, are obtained using *get*_*density()* and *get*_*potential()* methods (Lines 25, 27), respectively. The PyEmbed module has all of the methods needed to manage the density of both the reference system and the environment to finally compute the nonadditive embedding potential. Indeed, the *embed*_*eval* object is instantiated (Line 34) and the nonadditive embedding potential is evaluated on the numerical grid using *get*_*nad*_*pot* (Line 36), once the densities of both the active system and of the environment have been provided.

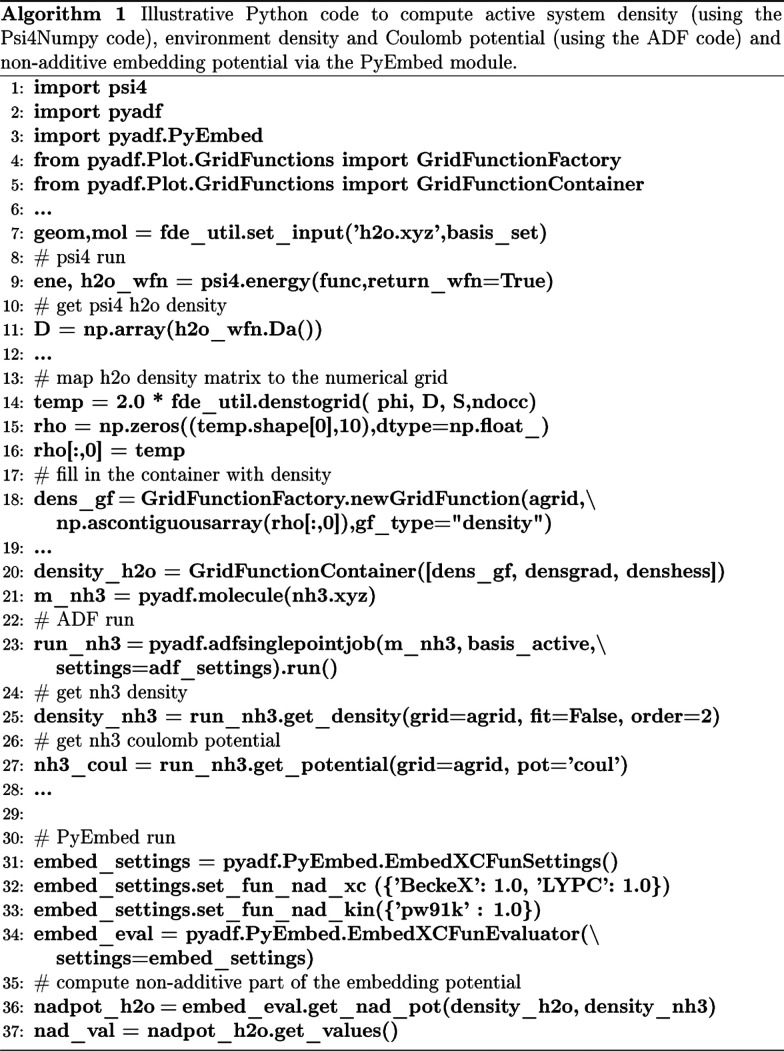



**Figure 1:**
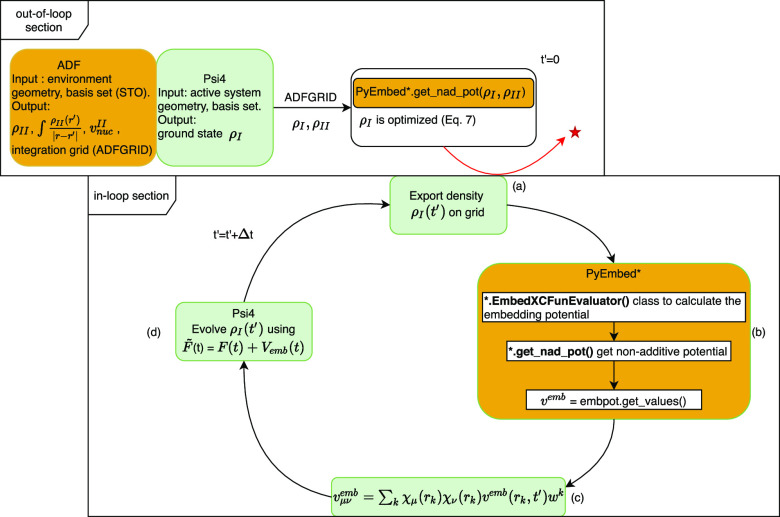
Working flowchart of the uFDE-RT-TDDFT. In the out-of-loop section, the density and electrostatic potential of the environment are obtained as grid functions. The active system density matrix is expressed as a grid function object and used to calculate the embedding potential. The active system density is optimized self-consistently according to eq 7. The red star and the arrow pointing at it symbolize that the out-of-loop blocks of tasks are involved only in the initial stage of the procedure. (a) The relaxed active density matrix is exported as a grid function. (b) PyEmbed classes are used to calculate the embedding potential. (c) The embedding potential is expressed on the finite basis set representation (GTOs). (d) The active density matrix is evolved according to the real-time-propagation scheme.

Algorithm 1 has well illustrated how we can utilize the classes provided by PyADF to obtain a very simple workflow in which we are able to manipulate quantities arising from Psi4Numpy. Thus, we are now in a position to draw the main lines of our uFDE-rt-TDDFT implementation, the Psi4-RT-PyEmbed code.^108^ In Figure 1, we present its pictorial workflow.

We start describing the out-of-loop section. First, the geometry and basis set of the environment are initialized (the orange leftmost block); thus, the ADF package provides, through a standalone single-point calculation, the electrostatic and nuclear potentials of the environment and its density ρ_II_ and a suitable integration grid for later use. At this stage, all of the basis sets and exchange-correlation functionals available in the ADF library can be used. In the next step, the green block, the geometry, and the basis set of the active system are parsed from the input and the ground-state density ρ_I_ is calculated using the Psi4Numpy-related methods. The right-pointing arrow, connecting the last block, sketches the mapping of the density matrix onto the real-space grid representation. The evaluation of ρ_I_(***r***) on the numerical grid is efficiently accomplished using the molecular orbitals (MOs), which requires the valuation of the localized basis functions at the grid points.

Finally, the PyEmbed module comes into play (the last block of the out-of-loop section); the real-space electron densities ρ_I_ and ρ_II_ serve as input for the *get*_*nad*_*pot()* method. Thus, the nonadditive kinetic and exchange potentials are obtained. The embedding potential is then calculated from its constituents (i.e., the environment electrostatic and nuclear potentials and the nonadditive contribution as detailed in eq 7) and evaluated at each grid point, *v*^emb^(***r***_*k*_). The embedding potential matrix representation in the active subsystem basis set, ***V***^emb^, is calculated numerically on the grid as
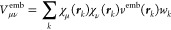
20where χ_μ_(***r***_*k*_) are the Gaussian-type basis set functions employed in the active systems (used in Psi4Numpy) evaluated at the grid point, ***r***_*k*_. In the above expression, *w*_*k*_ are specific integration weights.

In the case of an FDE-rt-TDDFT calculation, the electron density of the active system at the beginning of the propagation (*t*_0_ = 0, initial condition) is not the ground-state density of the isolated molecule but rather a polarized ground-state density. The latter is obtained through a self-consistent-field calculation in the presence of the embedding potential. We adopt the so-called split-SCF scheme as described in ref (114). It should be noted that the density matrix, corresponding to the optimized ρ_I_ electron density, is the input data for the green block (block a) of the in-loop section. The outgoing red arrow, connecting the out- and the in-loop branch of the diagram, indicates that the former is only involved in the early step of the procedure and it will no longer come into play during the time propagation. As mentioned, the optimized density matrix of the active system resulting from the SCF procedure, including the embedding potential, is the starting point for the real-time propagation. Whereupon, at each time step, we determine the embedding potential corresponding to the instantaneous active density (*v*^emb^[ρ_I_(*t*), ρ_II_]). Again, we need its mapping onto the real-space grid as shown in the first green box (box a). Then, we utilize the methods reported in the rectangular orange box (box b) to calculate the nonadditive part of the embedding potential at each grid point. Finally, we add the nonadditive (kinetic and exchange-correlation) potential to the electrostatic potential of the environment calculated again at each grid point. It should be noted that because the density of the environment is frozen, the corresponding electrostatic potential remains constant during the time propagation. In the next phase, its matrix representation in the localized Gaussian basis functions is obtained as in eq 20 (box c, in Figure 1). The active system is evolved (box d) using an effective time-dependent Kohn–Sham matrix, which contains the usual implicit and explicit time-dependent terms, respectively, (**J**[ρ_I_(*t*)] + **V**_xc_ [ρ_I_(*t*)]) and **v**_ext_(*t*), plus the time-dependent embedding potential (***V***^emb^[ρ_I_(*t*), ρ_II_]).

For the sake of completeness, the pseudocode needed to evolve the density using the second-order midpoint Magnus propagator is reported in the SI and relies on the methodology illustrated in Section 2.2. We refer the interested readers to our recent work on real-time propagation for further details.^37^

## 4 Results and Discussion

In the present section, we report a series of results mainly devoted to assessing the correctness of the uFDE-rt-TDDFT scheme. To the best of our knowledge, this implementation is the first available for localized basis sets. Since our implementation relies on the embedding strategies implemented in PyADF, it appears natural and appropriate to choose as a useful reference the uncoupled FDE-TDDFT scheme, based on the linear response^75,115^ and implemented in the ADF program package.^116^

### 4.1 Initial Validation and Numerical Stability

Before going into the details of the numerical comparison between our implementation and the FDE-TDDFT scheme based on the linear-response (ADF-LR) formalism, whether in combination with FDE (ADF-LR-FDE) or not, it is important to first assess the basis set dependence of the calculated excitation energies using the two different approaches. This preliminary study is mandatory because Psi4Numpy (Gaussians) and ADF (Slaters) employ different types of atom-centered basis functions. Due to this difference, perfect numerical agreement between the two implementations cannot be expected, but it is important to quantify the variability of our target observables (the excitation energies of a water molecule) with variations in the basis set.

To simulate the linear-response regime within our Psi4-rt, the electronic ground state of a water molecule, calculated in the absence of an external electric field, was perturbed by an analytic δ-function pulse with a strength of κ = 1.0 × 10^–5^ a.u. along the three directions, *x*, *y*, and *z*. The induced dipole moment was collected for 9000 time steps with a length of 0.1 a.u. per time step, corresponding to 21.7 fs of simulation. This time-dependent dipole moment was then Fourier-transformed to obtain the dipole strength function *S*(ω), according to eq 18 and the transition energies. The Fourier transform of the induced dipole moment was carried out by means of Padé approximants.^17,117^

As shown in Table 1, convergence can be observed with both Psi4-rt and ADF-LR, in particular for the first low-lying transitions (additional excitation energies are reported in the Supporting Information). For some of the higher energy transitions, the convergence is less prominent, indicating deficiencies in the smaller basis sets. We mention that the results obtained using our Psi4-rt implementation perfectly agree with those obtained using the TDDFT implementation based on linear-response implemented in the NWChem code, which uses the same Gaussian-type basis set (see Table S1 in the SI). Thus, we conclude that most of the deviations from the ADF-LR values can be ascribed to unavoidable basis set differences. A qualitatively similar pattern of differences is to be expected when including the environment effect within the FDE framework.

**Table 1:** Excitation Energies (in eV) Corresponding to the First Five Low-Lying Transitions of the Isolated Water Molecule^a^

excitation energy (eV)						
	Psi4-rt			ADF-LR		
	D	T	Q	D	T	Q
root 1	6.215	6.227	6.224	6.161	6.189	6.287
root 2	7.512	7.466	7.440	7.454	7.465	7.884
root 3	8.363	8.352	8.344	8.309	8.288	8.427
root 4	9.536	8.953	8.651	8.803	8.482	8.628
root 5	9.644	9.572	9.306	8.945	8.845	10.022

a Data obtained using TDDFT based on linear-response implemented in ADF (ADF-LR) and the real-time TDDFT implemented (Psi4-rt). The labels (D, T, Q) correspond to data obtained using the Gaussian-type basis sets aug-cc-pVXZ (X = D, T, Q) and Slater-type basis sets AUG-X′ (X′ = DZP, TZ2P, QZ4P), which are used in the Psi4-rt and ADF-LR codes, respectively (see the text for details).

To assess differences in the presence of an environment, we next tested our uFDE-rt-TDDFT results against ADF-LR-FDE ones. The target system is the water–ammonia adduct, in which the water molecule is the active system that is bound to an ammonia molecule, which plays the role of the embedding environment. In the Psi4-rt-PyEmbed case, we employed a contracted Gaussian aug-cc-pVXZ (X = D, T) basis set^118,119^ for the active system, whereas the basis set used in PyADF for the calculation of the environment frozen density (ammonia) and the embedding potential is the AUG-X′ (X′ = DZP, TZ2P) Slater-type set from the ADF library.^116^ The ADF-LR-FDE employs the AUG-X′ (X′ = DZP, TZ2P) basis sets from the same library. For the real-time propagation of the active system (water), in both the isolated and the embedded case, the BLYP^120,121^ exchange-correlation functional is used, while the Thomas–Fermi and LDA functionals^122,123^ have been employed for the nonadditive kinetic and nonadditive exchange-correlation potentials, respectively. The numerical results are reported in Table 2. Although, as expected, there is no quantitative agreement on the absolute value of the transitions, the shift Δ (*E*_iso._ – *E*_emb_) shows an acceptable agreement for the lowest transitions (for additional excitation energies, see the Supporting Information).

**Table 2:** Excitation Energies (in eV) Corresponding to the First Five Low-Lying Transitions of Both the Isolated and Embedded Water Molecules^a^

excitation energy (eV)						
	Psi4-rt-PyEmbed			ADF-LR-FDE		
	isolated	emb.	Δ	isolated	emb.	Δ
(a) double-zeta calculations						
root 1	6.215	5.817	0.398	6.161	5.687	0.474
root 2	7.512	6.694	0.818	7.454	6.578	0.876
root 3	8.363	7.892	0.470	8.309	7.782	0.527
root 4	9.536	8.768	0.768	8.803	8.336	0.467
root 5	9.644	9.186	0.458	8.945	8.422	0.523
(b) triple-zeta calculations						
root 1	6.227	5.796	0.430	6.189	5.689	0.500
root 2	7.466	6.573	0.893	7.465	6.559	0.905
root 3	8.352	7.848	0.503	8.288	7.734	0.554
root 4	8.953	8.560	0.393	8.482	7.969	0.513
root 5	9.572	8.625	0.948	8.845	8.318	0.527

a In the embedded water molecule, an ammonia molecule is used as the environment. Data have been obtained using our new Psi4-rt-PyEmbed implementation and the reference ADF-LR-FDE implementation with (a) aug-cc-pVDZ and AUG-DZP basis sets and (b) aug-cc-pVTZ and AUG-TZ2P basis sets (see the text for details). The shift Δ (*E*_iso._ – *E*_emb_) in the transition energies due to the embedding environment is also reported.

From these results, we conclude that our implementation is both stable and numerically correct, with differences between the methods explainable by the intrinsic basis set differences.

**Figure 2:**
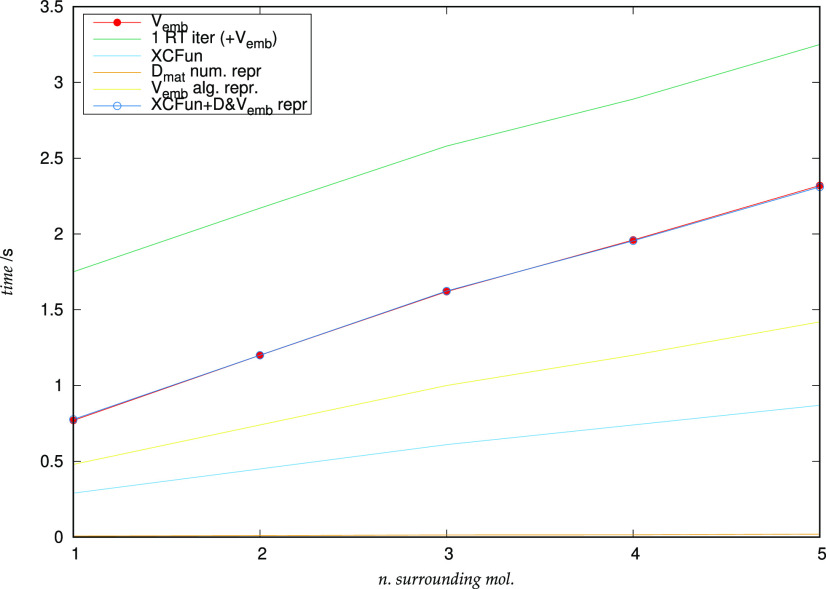
Time needed for different tasks vs number of surrounding molecules.

### 4.2 Water-in-Water Test Case

**Figure 3:**
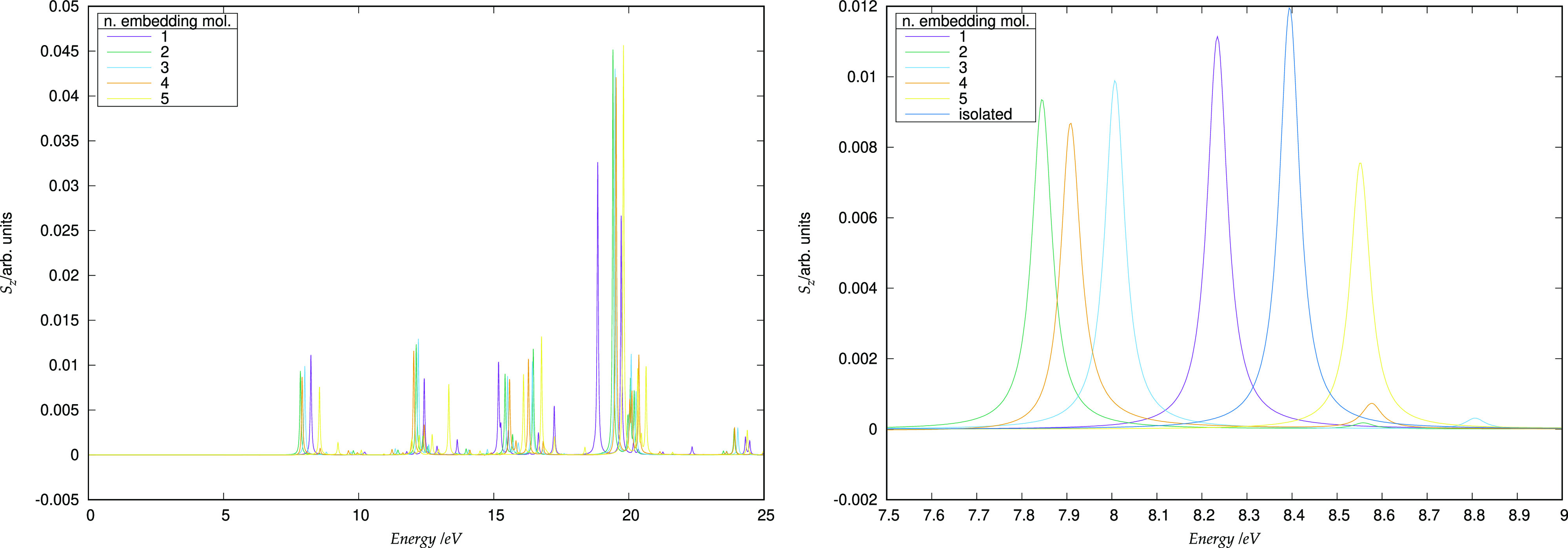
Dipole strength function *S*_*z*_ of the water cluster as a function of the number of surrounding molecules (left panel). Right panel: detailed representation of the low-lying transition. The peak corresponding to the isolated molecule is reported for comparison.

To provide a further test of our implementation, we also computed the absorption spectra of a water molecule embedded in a water cluster of increasing size. The geometries of the different water clusters are taken from refs (124, 125), which correspond to one snapshot taken from an MD simulation. Different cluster models were taken into consideration, by progressive addition of surrounding water molecules (from 1 to 5 molecules) to the single active water molecule. For the active system water molecule propagation in Psi4-rt-PyEmbed, we use the aug-cc-pVDZ basis set, while for the environment, computed using the ADF code, we use the AUG-DZP basis set. In both cases, we use the BLYP^120,121^ exchange-correlation functional, while for the nonadditive kinetic and nonadditive exchange-correlation terms in the generation of the embedding potential, the Thomas–Fermi and LDA functionals are used, respectively. In each case, we use 9000 time steps of propagation, which correspond to a simulation of ≈22 fs (time step of 0.1 a.u.). The corresponding dipole strength functions (*S*_*z*_(ω) = 2ω/(3π)Im[α_*zz*_(ω)]) along the *z*-direction are reported in Figure 3. Upon the increase of the cluster dimension, the lowest-lying transition shifts within a range of about 1 eV, which is consistent with the results for liquid water.^126^ It is worth noting that, for this specific transition, many-body excitonic effects (included via the full coupled FDE-rt-TDDFT scheme by Pavanello et al.) are negligible for the energy but are found to be very important to reproduce its spectral intensity.^126^ These results give confidence in the numerical stability of the propagation when the number of molecules in the environment is increased.

The systematic increase of the size of the environment makes it possible to also consider the actual computational scaling of the Psi4-rt-PyEmbed code for this case. To show this scaling, we carried out a single time step of the real-time propagation and broke down the computational cost into those of the different steps in the workflow, as reported in Figure 1.

In Table 3 and Figure 2, we report how the time for the embedding potential calculation is distributed over the different tasks when the number of surrounding water molecules increases from one to five. It is interesting to note that the time needed to evaluate the embedding potential increases almost linearly for the limited number of water molecules considered here. The standard real-time iteration time (corresponding to the isolated water molecule) takes less than 1 s and shows up as a fixed cost in the increasing computation time, while the time spent in the embedding part is dominated by the evaluation of the matrix representation for the active subsystem (e.g., step c) of Figure 1 (see for instance the *t*^c^ column of Table 3). The time spent in this evaluation depends on the number of numerical integration points used to represent the potential and can be reduced using special grids for embedding purposes once the environment is large enough.

**Table 3:** Time Usage in Seconds

	*t*^a^	*t*^b^	*t*^c^	*t*^d^	*t*^e^
1	0.007	0.29	0.48	0.77	1.75
2	0.01	0.45	0.74	1.20	2.17
3	0.014	0.61	1.0	1.62	2.58
4	0.015	0.74	1.2	1.96	2.89
5	0.02	0.87	1.42	2.32	3.25

a Density on the grid (through MOs).

b XCFun (nonadditive potential calculation).

c *V*_emb_ projection onto the basis set.

d Total time for *V*_emb_ evaluation.

e Total time for an rt-iteration.

### 4.3 Acetone-in-Water Test Case

**Figure 4:**
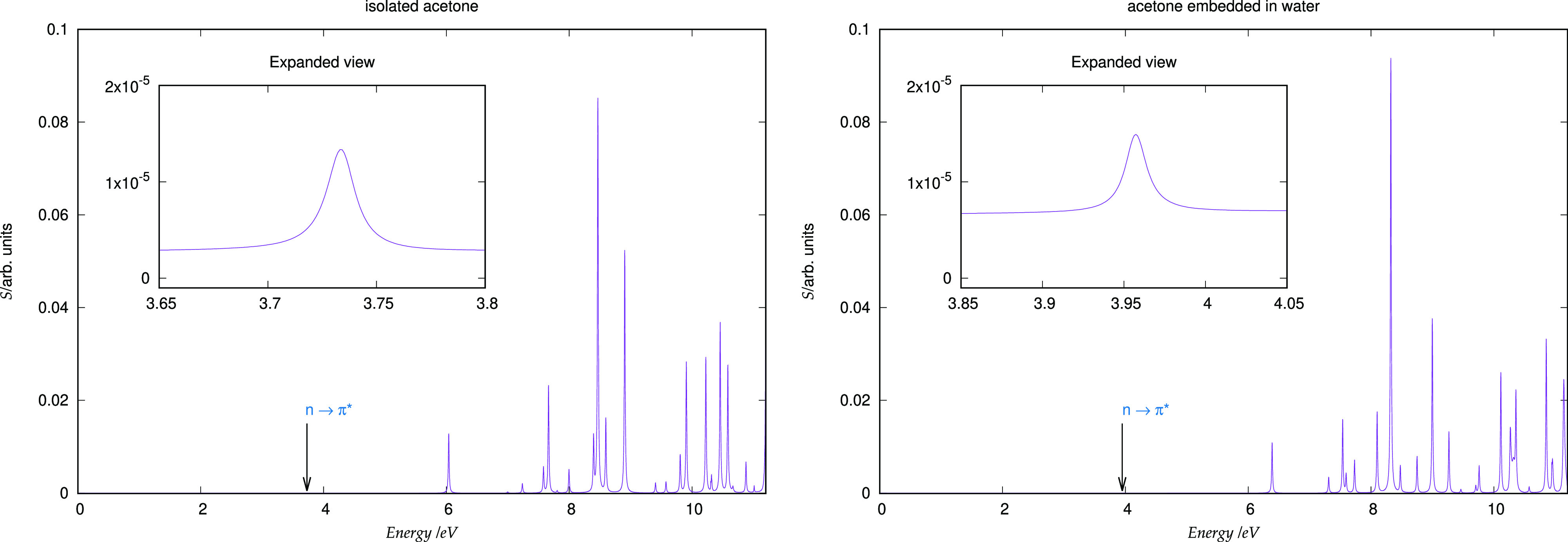
Absorption spectrum of isolated acetone (left panel) and embedded acetone in a water cluster (right panel).

As a further test of the numerical stability of accuracy of the method, we investigated the *n* → π* transition in the acetone molecule, both isolated and using an explicit water cluster to model solvation. To assess the shift due to the embedding potential, we calculated the absorption spectrum of the isolated molecule at the same geometry it has in the cluster model. The geometry for the solvated acetone system was taken from ref (91), corresponding to one snapshot from an MD simulation, where the acetone is surrounded by an environment consisting of 56 water molecules. The uFDE-rt-TDDFT calculation was obtained specifying in our Psi4-rt-PyEmbed framework all of the computational details. In particular, the frozen density of the environment is obtained from a ground-state calculation using ADF in combination with the PBE functional and the DZP basis set, while for acetone, we employ the BLYP functional and the Gaussian def2-svp basis set using the Psi4-rt code. The nonadditive kinetic and exchange-correlation terms of the embedding potential are calculated using the Thomas–Fermi and LDA functionals, respectively. For the isolated acetone, the *n* → π* transition is found at 3.73 eV, whereas for the embedded molecule, it is located at 3.96 eV. The full absorption spectrum is reported in Figure 4.

It is worth noting that, due to its low intensity, this transition is particularly challenging for a real-time propagation framework. To obtain a spectrum up to 11 eV, we carried out a simulation consisting of 20 000 time steps and lasting 2000 a.u. (48 fs). This relatively long simulation time demonstrates the numerical stability of the approach and its implementation.

As an overall check of our implementation, we compare the shift of the *n* → π* transition observed between isolated acetone and acetone embedded in a water cluster obtained using both of our Psi4-rt-PyEmbed and ADF-LR-FDE methods. The active system response was calculated at the BLYP level of theory, while the Thomas–Fermi and LDA functionals were employed for the nonadditive kinetic and exchange-correlation terms, respectively, of the embedding potential in the ADF-LR-FDE calculation. As one can observe by looking at the values reported in Table 4, we obtain a good agreement in the absolute values, for both isolated and embedded acetone, and the computed shift is likewise in rather good agreement.

**Table 4:** Isolated and Embedded in a Water Cluster Acetone *n* → π* Transition, Reported for Both ADF-LR-FDE and Our Psi4-rt-PyEmbed Code

	iso. (eV)	emb. (eV)	Δ*E* (eV)
Psi4-rt-PyEmbed	3.734	3.958	0.225
ADF-LR-FDE	3.793	3.975	0.182

### 4.4 FDE-rt-TDDFT in the Nonlinear Regime

A specificity in the real-time approach is that the evolution of the electron density can be driven by a real-valued electric field whose shape can be explicitly modulated. Realistic laser fields can be modeled by a *sine* function of ω_0_ frequency using any physically meaningful enveloping function. Using an explicit external field is a key tool in the optical control theory; furthermore, it is possible, employing a high-intensity field, to study phenomena beyond linear response, i.e., hyperpolarizability coefficients and high harmonic generation in molecules. The latter point will be detailed in the following section.

In this section, we demonstrate that the uFDE-rt-TDDFT scheme gives stable numerical results not only in the perturbative regime, as shown above, but also in the presence of intense fields. Physically meaningful laser fields are adequately represented by sinusoidal pulse of the form *E*(*t*) = *f*(*t*) sin(ω_0_*t*), where ω_0_ is the carrier frequency. In this work, we employ a cos^2^ shape for the envelope function^127^
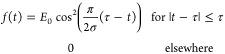
where τ is the width of the field envelope. We calculated the response of H_2_O embedded in a water cluster model made of five water molecules (all of the details about the geometry have been reported in a previous section) to a cos^2^-shaped laser field with carrier frequency ω_0_ = 1.55 eV (analogous to a Ti:sapphire laser), intensity *I* = 1.02 × 10^14^ W cm^–2^ (which corresponds to a field *E* = 0.054 a.u.), and a duration of 20 optical cycles. Each cycle lasts 2π/ω_0_, and the overall pulse spans over 2250.0 a.u. (i.e., 54 fs). The field was chosen along the molecular symmetry axis (*z*), and the 6-311++G** basis set and the B3LYP functional were used. The propagation was carried out for a total time of 3500 a.u. without any numerical instabilities.

**Figure 5:**
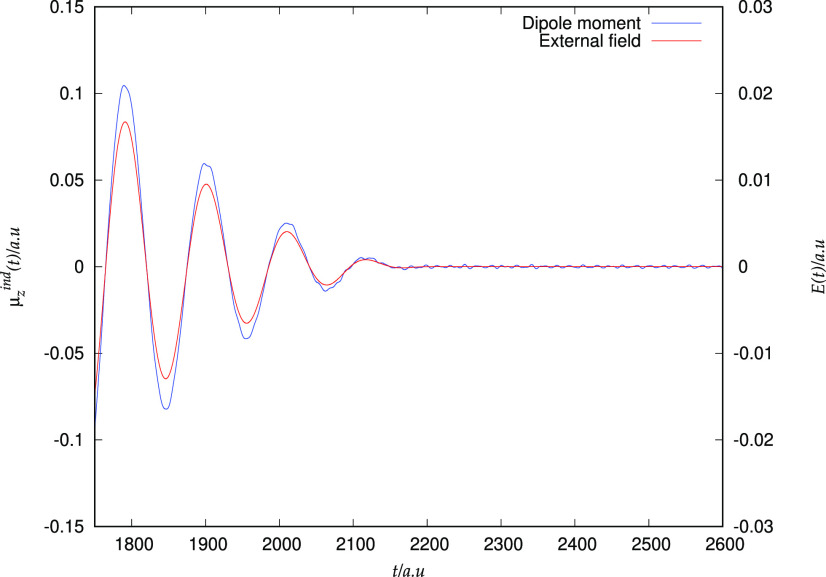
Induced dipole moment in the H_2_O molecule. The representation of the external field is also reported as a green line.

**Figure 6:**
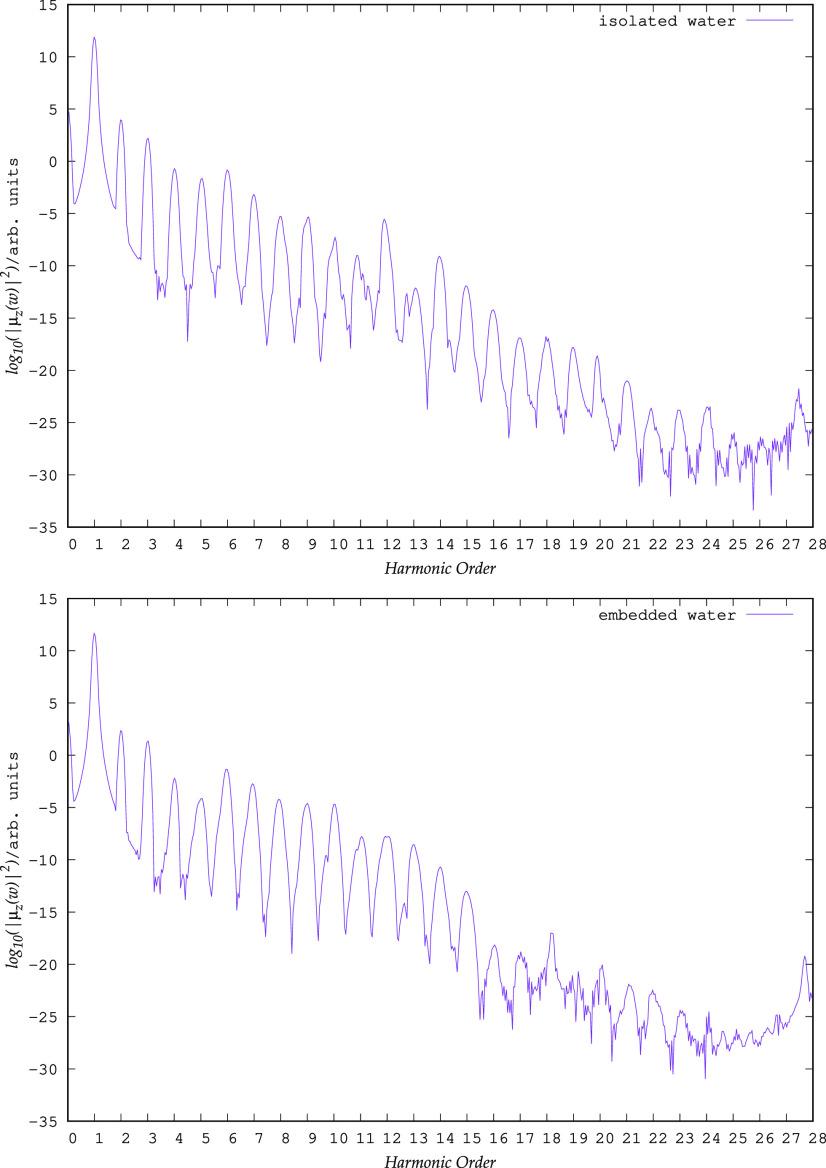
Upper panel: emission spectrum of the isolated water molecule. Lower panel: emission spectrum of the same water molecule embedded in the (H_2_O)_5_ cluster.

As shown in Figure 5, the induced dipole does not follow the applied field adiabatically when a strong field is applied; especially in the last few optical cycles, strong diabatic effects are clearly present. These effects lead to the presence of a residual dipole oscillation. Following a previous work on high harmonic generation (HHG) in the H_2_ molecule,^127^ we extracted the high-order harmonic intensities via the Fourier transform of the laser-driven induced dipole moment (neglecting the remaining part, i.e., for *t* larger than τ, i.e., 2250 a.u. in the present simulation) as
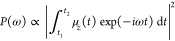
21In Figure 6, we report the base-10 logarithm of the spectral intensity for the embedded water molecule and we compare it to the HHG of the isolated water calculated that has the same geometry it has in the cluster model. In the case of the isolated water molecule, we were able to observe relatively well-defined peaks up to the 21st harmonics. We mention that this finding qualitatively agrees with data obtained by Sun et al.^20^ (see Figure 3 of ref (20)).

An important parameter in the analysis of the HHG spectrum is the value of the energy cutoff (*E*_cutoff_), which is related to the maximum number of high harmonics (*N*_max_ ≈ *E*_cutoff_/ω_0_). In a semiclassical formulation,^128^ which, among others, assumes that only a single electron is active for HHG, *E*_cutoff_ ≈ *I*_*p*_ + 3.17*U*_*p*_, where *I*_*p*_ is the ionization potential of the system and *U*_*p*_ () is the ponderomotive energy in the laser field of strength *E* and frequency ω_0_.^128^ In the case of molecular systems, the HHG spectra present more complex features and the above formula is not strictly valid. With the laser parameters used here (*E* = 0.054 a.u., ω_0_ = 0.05696 a.u.) and the experimental ionization potential of H_2_O (*I*_*p*_ = 0.4637 a.u.), the above formula predicts an *E*_cutoff_ value of 1.17601 a.u. (*N*_max_ at about the 21st harmonic), which is remarkably consistent with the HHG spectra we observed here.

For the water molecule embedded in the cluster, the same boundary can be approximately found corresponding to the 16th harmonic. The peaks at higher energies have a very small intensity and are much less resolved above the 16th harmonic. The flattening of the HHG intensity pattern is therefore solely due to the introduction of the embedding potential of the surrounding cluster. The latter is consistent with a shift toward lower ionization energy passing from the free water molecule to a small water cluster observed experimentally.^129^

### 4.5 Computational Constraints

Before concluding this work, it may be interesting to put forward some assessments in terms of time statistics to be used as a basis for optimizing the computation time and speed-up any uFDE-rt-TDDFT calculations. We used a water–ammonia complex as a general test-case, where the geometry of the adduct was taken from ref (130) and water is the active subsystem.

In the real-time framework, the embedding potential is, evidently, an implicit time-dependent quantity. Since in the uncoupled FDE framework the density of the environment is kept frozen, the embedding potential depends on time only through the relatively small contributions given by the exchange-correlation and kinetic nonadditive terms, which in turn depend on time only through the density of the active subsystem. The electrostatic potential, due to the frozen electron density and nuclear charges of the environment, is the leading term in the overall potential. Thus, it may be reasonable to choose a longer time step to update the embedding potential, which weakly varies in time.

**Figure 7:**
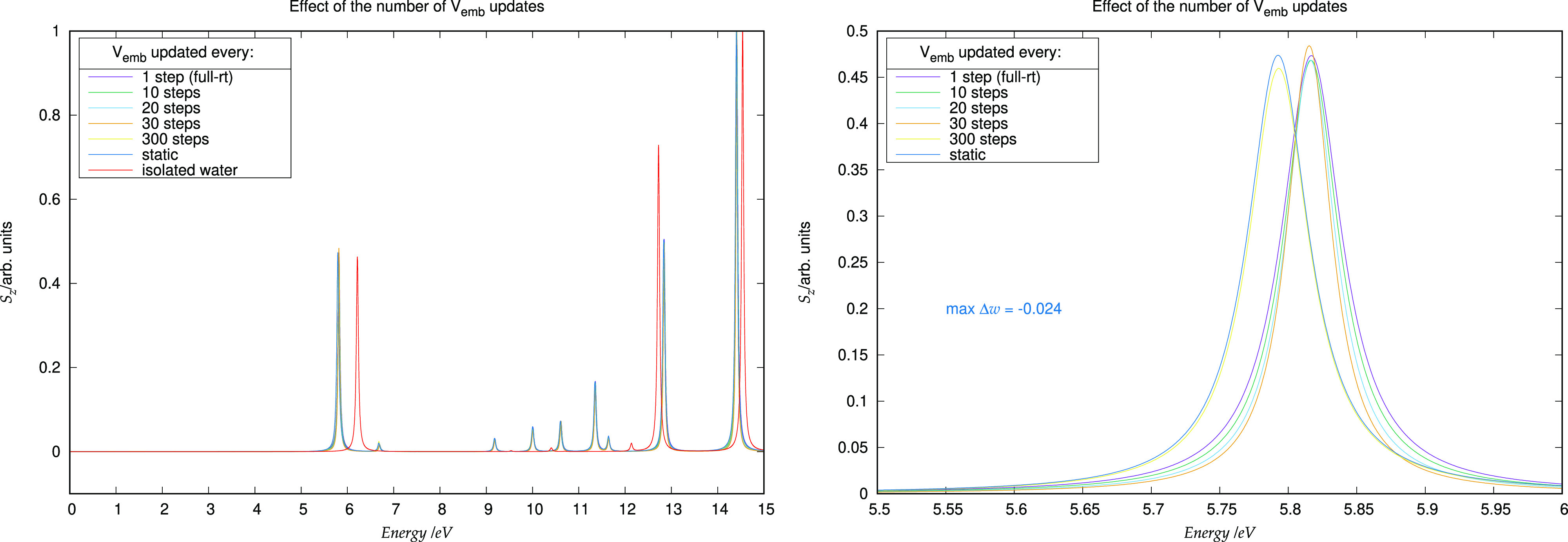
Left: frequency shift in the *S*_*z*_ function due to the increasing rate of update of the embedding potential. The peaks corresponding to the isolated water molecule are also reported as red trace. Right: expanded view of the homo–lumo transition.

To investigate such a possible speed-up, we carried out different simulations in which the time interval of the embedding potential updating is progressively increased. The results are reported in Table 5. Of course, as the number of time steps between consecutive updates is increased (i.e., the embedding potential is updated less often), the total time needed to perform the full simulation goes down, as the time spent in computing the embedding potential decreases. The update rate of the embedding potential during the propagation affects to some extent the position of the peaks in the absorption spectrum. As can be seen in Figure 7, the different traces corresponding to dipole strength functions calculated with different update rates do not differ significantly and tend to coalesce as the number of time steps between consecutive updates decreases below 30 time steps. In particular, in the case of the lowest-energy transition, the energy shift corresponding to a quite long update period (roughly 300 time steps) is on the order of 0.02 eV.

**Table 5:** Time in Seconds as a Function of the Number *n* of Time Steps between Consecutive Updates of the Embedding Potential

*t*^a^	*t*^b^	*t*^c^	*n*
0.87		94.84	inf (static)
0.87	2.59	97.52	30
0.85	4.32	99.26	20
0.86	8.56	103.31	10
0.86	85.67	180.97	1

a Time for *V*_emb_ evaluation.

b Total time for *V*_emb_ evaluation in the propagation.

c Total time needed for 100 real-time iterations.

**Figure 8:**
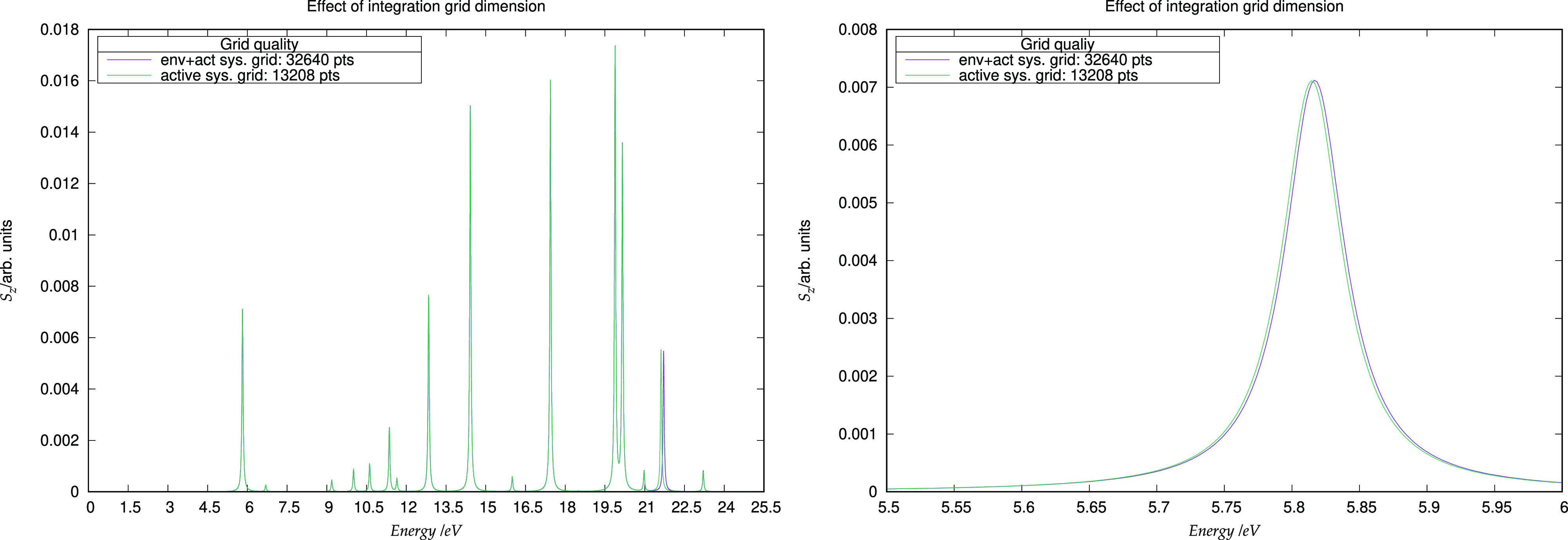
Comparison of the *S*_*z*_ dipole strength function obtained with two different integration grids. The violet trace corresponds to the full supramolecular integration grid, while the green trace corresponds to the active system grid. Right: expanded view of the lowest-lying transition.

We also reported the partition between different tasks of the time needed for the calculation of the embedding potential in Table 6. As seen before, the calculation of the embedding potential is largely dominated by the projection to the basis set of the embedding potential from the numerical-grid representation. Therefore, some preliminary tests in reducing the number of grid points were carried out, and the results are presented in Figure 8. It can be seen that there is no significant modification in the peak positions due to the use of a coarser integration grid: the overall spectrum is essentially stable and no artifacts are introduced.

**Table 6:** Time Usage in Seconds

*t*^a^	*t*^b^	*t*^c^	*t*^d^
0.01	0.33	0.53	0.87

a Density on the grid (through MOs).

b XCFun (nonadditive potential calculation).

c *V*_emb_ projection onto the basis set.

d Total time for *V*_emb_ evaluation.

We furthermore note the possibility of using a small grid localized solely on the active system by utilizing the fact that the embedding potential is projected on the localized basis set functions of the active system (see eq 20), which makes it possible to neglect points on which these functions have a small value.

## 5 Conclusions and Perspectives

In this work, we focused on the implementation of the frozen-density embedding scheme in the real-time TDDFT. We integrated the Psi4Numpy real-time module we recently developed within the PyADF framework. We devised a real-time FDE scheme in which the active density evolves in the presence of the embedding potential. This implementation relies on a multiscale approach, since the embedding potential is calculated by means of PyADF, while the propagation is carried out by Psi4Numpy. We tested the implementation on a simple water cluster, showing that the time needed for the propagation scales linearly with cluster size. We studied many low-lying transitions in the case of a water molecule embedded in ammonia, and we showed that the shift of excitation energies with respect to the isolated water molecule is in good agreement with the results obtained using linear-response FDE-TDDFT implemented in ADF. Finally, we tackled a challenging case for rt-TDDFT by computing the lowest-energy transition of acetone, which features an extremely low intensity. The corresponding signal can be identified in the computed spectrum, and we evaluated the solvatochromic shift due to the presence of a surrounding water cluster. We obtained a frequency shift of 0.225 eV, close to the reference value, 0.182 eV, from LR-FDE-TDDFT as implemented in ADF. The scheme we developed proved to be reliable also in the case of propagation in the nonlinear regime. As a demonstration, we perturbed with a strong electric field a water molecule surrounded by five water molecules acting as a frozen environment. Numerically stable induced dipole moment and the corresponding emission spectrum were obtained.

Finally, we would like to state that the present work provides an excellent framework for future developments. It is, for instance, possible and desirable to optimize the embedding potential construction. In our implementation (i.e., Psi4-rt-PyEmbed), the projection onto the basis set of the embedding potential from the numerical-grid representation dominates the computational burden. The change of the embedding potential matrix in time (i.e., the difference at two consecutive time steps) depends on the relatively small contributions given by the exchange-correlation and kinetic nonadditive terms. Significant improvement could be achieved by exploiting the sparsity of the matrix corresponding to that difference. Moreover, the use of a smaller integration grid would probably further improve the procedure. Last but not the least, the effect of relaxation of the environment has to be investigated. In our uncoupled FDE-rt-TDDFT scheme, we were able to study local transitions within a given subsystem and particularly those of the active system under the influence of the embedding potential due to the frozen environment. Thus, we neglect transitions involving the environment and those due to the couplings of the subsystems. Relaxing the environment can be crucial both in the linear-response framework, to recover supramolecular excitations, and in the nonlinear regime, where a polarizable environment could heavily affect the hyperpolarizabilities of the target system. The limit of the uncoupled FDE scheme can be overcome by carrying out a simultaneous propagation of subsystems,^70^ and the computational framework developed in the present work represents an important step in that direction.

## Supporting Information Available

Supporting information reports the pseudocode used to propagate the density matrix in time, excitation energies in isolated water (Psi4-RT and NWChem-LR vs ADF-LR), basis set convergence of excitation energies with both Psi4-rt and ADF-LR, and numerical comparison between excitation energies of water embedded in ammonia obtained from uFDE-rt-TDDFT and ADF-LR-FDE (PDF)

## References

[ref1] Hardin B. E.; Hoke E. T.; Armstrong P. B.; Yum J.-H.; Comte P.; Torres T.; Fréchet J. M.; Nazeeruddin M. K.; Grätzel M.; McGehee M. D. Increased light harvesting in dye-sensitized solar cells with energy relay dyes. Nat. Photonics 2009, 3, 406 10.1038/nphoton.2009.96.

[ref2] Hagfeldt A.; Boschloo G.; Sun L.; Kloo L.; Pettersson H. Dye-sensitized solar cells. Chem. Rev. 2010, 110, 6595–6663. 10.1021/cr900356p. 20831177

[ref3] Salières P.; Le Déroff L.; Auguste T.; Monot P.; d’Oliveira P.; Campo D.; Hergott J.-F.; Merdji H.; Carré B. Frequency-Domain Interferometry in the XUV with High-Order Harmonics. Phys. Rev. Lett. 1999, 83, 5483–5486. 10.1103/PhysRevLett.83.5483.

[ref4] Paul P. M.; Toma E. S.; Breger P.; Mullot G.; Augé F.; Balcou P.; Muller H. G.; Agostini P. Observation of a Train of Attosecond Pulses from High Harmonic Generation. Science 2001, 292, 1689–1692. 10.1126/science.1059413. 11387467

[ref5] Bass M.; Franken P. A.; Ward J. F.; Weinreich G. Optical Rectification. Phys. Rev. Lett. 1962, 9, 446–448. 10.1103/PhysRevLett.9.446.

[ref6] Kadlec F.; Kužel P.; Coutaz J.-L. Study of terahertz radiation generated by optical rectification on thin gold films. Opt. Lett. 2005, 30, 1402–1404. 10.1364/OL.30.001402. 15981547

[ref7] Keldysh L. V. Multiphoton ionization by a very short pulse. Physics-Uspekhi 2017, 60, 1187–1193.

[ref8] Eberly J.; Javanainen J.; Rzażewski K. Above-threshold ionization. Phys. Rep. 1991, 204, 331–383. 10.1016/0370-1573(91)90131-5.

[ref9] Gallmann L.; Cirelli C.; Keller U. Attosecond Science: Recent Highlights and Future Trends. Annu. Rev. Phys. Chem. 2012, 63, 447–469. 10.1146/annurev-physchem-032511-143702. 22404594

[ref10] Ramasesha K.; Leone S. R.; Neumark D. M. Real-Time Probing of Electron Dynamics Using Attosecond Time-Resolved Spectroscopy. Annu. Rev. Phys. Chem. 2016, 67, 41–63. 10.1146/annurev-physchem-040215-112025. 26980312

[ref11] Attar A. R.; Bhattacherjee A.; Pemmaraju C. D.; Schnorr K.; Closser K. D.; Prendergast D.; Leone S. R. Femtosecond x-ray spectroscopy of an electrocyclic ring-opening reaction. Science 2017, 356, 54–59. 10.1126/science.aaj2198. 28386006

[ref12] Wolf T. J. A.; Sanchez D. M.; Yang J.; Parrish R. M.; Nunes J. P. F.; Centurion M.; Coffee R.; Cryan J. P.; Ghr M.; Hegazy K.; Kirrander A.; Li R. K.; Ruddock J.; Shen X.; Vecchione T.; Weathersby S. P.; Weber P. M.; Wilkin K.; Yong H.; Zheng Q.; Wang X. J.; Minitti M. P.; Martinez T. J. The photochemical ring-opening of 1,3-cyclohexadiene imaged by ultrafast electron diffraction. Nat. Chem. 2019, 11, 504–509. 10.1038/s41557-019-0252-7. 30988415

[ref13] Ruddock J. M.; Zotev N.; Stankus B.; Yong H.; Bellshaw D.; Boutet S.; Lane T. J.; Liang M.; Carbajo S.; Du W.; Kirrander A.; Minitti M.; Weber P. M. Simplicity Beneath Complexity: Counting Molecular Electrons Reveals Transients and Kinetics of Photodissociation Reactions. Angew. Chem., Int. Ed. 2019, 131, 6437–6441. 10.1002/ange.201902228. 30866169

[ref14] Kim K. H.; Kim J. G.; Nozawa S.; Sato T.; Oang K. Y.; Kim T. W.; Ki H.; Jo J.; Park S.; Song C.; Sato T.; Ogawa K.; Togashi T.; Tono K.; Yabashi M.; Ishikawa T.; Kim J.; Ryoo R.; Kim J.; Ihee H.; Adachi S.-i. Direct observation of bond formation in solution with femtosecond X-ray scattering. Nature 2015, 518, 385–389. 10.1038/nature14163. 25693570

[ref15] Sharifi M.; Kong F.; Chin S. L.; Mineo H.; Dyakov Y.; Mebel A. M.; Chao S. D.; Hayashi M.; Lin S. H. Experimental and Theoretical Investigation of High-Power Laser Ionization and Dissociation of Methane. J. Phys. Chem. A 2007, 111, 9405–9416. 10.1021/jp074053f. 17764161

[ref16] Zigo S.; Le A.-T.; Timilsina P.; Trallero-Herrero C. A. Ionization study of isomeric molecules in strong-field laser pulses. Sci. Rep. 2017, 7, 42149 10.1038/srep42149. 28186110 PMC5301495

[ref17] Goings J. J.; Lestrange P. J.; Li X. Real-time time-dependent electronic structure theory. Wiley Interdiscip. Rev.: Comput. Mol. Sci. 2018, 8, e1341 10.1002/wcms.1341.

[ref18] Ekström U.; Visscher L.; Bast R.; Thorvaldsen A. J.; Ruud K. Arbitrary-Order Density Functional Response Theory from Automatic Differentiation. J. Chem. Theory Comput. 2010, 6, 1971–1980. 10.1021/ct100117s. 26615926

[ref19] Rosa M.; Gil G.; Corni S.; Cammi R. Quantum optimal control theory for solvated systems. J. Chem. Phys. 2019, 151, 194109 10.1063/1.5125184. 31757146

[ref20] Sun J.; Song J.; Zhao Y.; Liang W.-Z. Real-time propagation of the reduced one-electron density matrix in atom-centered Gaussian orbitals: Application to absorption spectra of silicon clusters. J. Chem. Phys. 2007, 127, 234107 10.1063/1.2805396. 18154375

[ref21] Li X.; Smith S. M.; Markevitch A. N.; Romanov D. A.; Levis R. J.; Schlegel H. B. A time-dependent Hartree-Fock approach for studying the electronic optical response of molecules in intense fields. Phys. Chem. Chem. Phys. 2005, 7, 233–239. 10.1039/B415849K. 19785143

[ref22] Eshuis H.; Balint-Kurti G. G.; Manby F. R. Dynamics of molecules in strong oscillating electric fields using time-dependent Hartree-Fock theory. J. Chem. Phys. 2008, 128, 114113 10.1063/1.2850415. 18361560

[ref23] Theilhaber J. Ab initio simulations of sodium using time-dependent density-functional theory. Phys. Rev. B 1992, 46, 12990–13003. 10.1103/PhysRevB.46.12990. 10003338

[ref24] Yabana K.; Bertsch G. F. Time-dependent local-density approximation in real time. Phys. Rev. B 1996, 54, 4484–4487. 10.1103/PhysRevB.54.4484. 9986402

[ref25] Takimoto Y.; Vila F.; Rehr J. Real-time time-dependent density functional theory approach for frequency-dependent nonlinear optical response in photonic molecules. J. Chem. Phys. 2007, 127, 154114 10.1063/1.2790014. 17949139

[ref26] Andrade X.; Strubbe D.; De Giovannini U.; Larsen A. H.; Oliveira M. J.; Alberdi-Rodriguez J.; Varas A.; Theophilou I.; Helbig N.; Verstraete M. J.; Stella L.; Nogueira F.; Aspuru-Guzik A.; Castro A.; Marques M. A. L.; Rubio A. Real-space grids and the Octopus code as tools for the development of new simulation approaches for electronic systems. Phys. Chem. Chem. Phys. 2015, 17, 31371–31396. 10.1039/C5CP00351B. 25721500

[ref27] Schleife A.; Draeger E. W.; Kanai Y.; Correa A. A. Plane-wave pseudopotential implementation of explicit integrators for time-dependent Kohn-Sham equations in large-scale simulations. J. Chem. Phys. 2012, 137, 22A546 10.1063/1.4758792. 23249083

[ref28] Giannozzi P.; Baseggio O.; Bonfà P.; Brunato D.; Car R.; Carnimeo I.; Cavazzoni C.; de Gironcoli S.; Delugas P.; Ferrari Ruffino F.; Ferretti A.; Marzari N.; Timrov I.; Urru A.; Baroni S. Quantum ESPRESSO toward the exascale. J. Chem. Phys. 2020, 152, 154105 10.1063/5.0005082. 32321275

[ref29] Genova A.; Ceresoli D.; Krishtal A.; Andreussi O.; DiStasio R. A. Jr.; Pavanello M. eQE: An open-source density functional embedding theory code for the condensed phase. Int. J. Quantum Chem. 2017, 117, e25401 10.1002/qua.25401.

[ref30] Liang W.; Chapman C. T.; Li X. Efficient first-principles electronic dynamics. J. Chem. Phys. 2011, 134, 184102 10.1063/1.3589144. 21568492

[ref31] Morzan U. N.; Ramírez F. F.; Oviedo M. B.; Sánchez C. G.; Scherlis D. A.; Lebrero M. C. G. Electron dynamics in complex environments with real-time time dependent density functional theory in a QM-MM framework. J. Chem. Phys. 2014, 140, 164105 10.1063/1.4871688. 24784251

[ref32] Lopata K.; Govind N. Modeling Fast Electron Dynamics with Real-Time Time-Dependent Density Functional Theory: Application to Small Molecules and Chromophores. J. Chem. Theory Comput. 2011, 7, 1344–1355. 10.1021/ct200137z. 26610129

[ref33] Nguyen T. S.; Parkhill J. Nonadiabatic Dynamics for Electrons at Second-Order: Real-Time TDDFT and OSCF2. J. Chem. Theory Comput. 2015, 11, 2918–2924. 10.1021/acs.jctc.5b00262. 26575729

[ref34] Zhu Y.; Herbert J. M. Self-consistent predictor/corrector algorithms for stable and efficient integration of the time-dependent Kohn-Sham equation. J. Chem. Phys. 2018, 148, 044117 10.1063/1.5004675. 29390834

[ref35] Repisky M.; Konecny L.; Kadek M.; Komorovsky S.; Malkin O. L.; Malkin V. G.; Ruud K. Excitation energies from real-time propagation of the four-component Dirac-Kohn-Sham equation. J. Chem. Theory Comput. 2015, 11, 980–991. 10.1021/ct501078d. 26579752

[ref36] Goings J. J.; Kasper J. M.; Egidi F.; Sun S.; Li X. Real time propagation of the exact two component time-dependent density functional theory. J. Chem. Phys. 2016, 145, 104107 10.1063/1.4962422. 27634251

[ref37] De Santis M.; Storchi L.; Belpassi L.; Quiney H. M.; Tarantelli F. PyBERTHART: ARelativistic Real-Time Four-Component TDDFT Implementation Using Prototyping Techniques Based on Python. J. Chem. Theory Comput. 2020, 16, 2410–2429. 10.1021/acs.jctc.0c00053. 32101419

[ref38] Storchi L.; De Santis M.; Belpassi L. PyBertha project git. https://github.com/lstorchi/pybertha.

[ref39] Smith D. G. A.; Burns L. A.; Sirianni D. A.; Nascimento D. R.; Kumar A.; James A. M.; Schriber J. B.; Zhang T.; Zhang B.; Abbott A. S.; Berquist E. J.; Lechner M. H.; Cunha L. A.; Heide A. G.; Waldrop J. M.; Takeshita T. Y.; Alenaizan A.; Neuhauser D.; King R. A.; Simmonett A. C.; Turney J. M.; Schaefer H. F.; Evangelista F. A.; DePrince A. E.; Crawford T. D.; Patkowski K.; Sherrill C. D. Psi4NumPy: An Interactive Quantum Chemistry Programming Environment for Reference Implementations and Rapid Development. J. Chem. Theory Comput. 2018, 14, 3504–3511. 10.1021/acs.jctc.8b00286. 29771539

[ref40] Belpassi L.; Storchi L.; Quiney H. M.; Tarantelli F. Recent Advances and Perspectives in Four-Component Dirac-Kohn-Sham Calculations. Phys. Chem. Chem. Phys. 2011, 13, 12368–12394. 10.1039/c1cp20569b. 21670843

[ref41] Belpassi L.; Tarantelli F.; Sgamellotti A.; Quiney H. M. Electron density fitting for the Coulomb problem in relativistic density-functional theory. J. Chem. Phys. 2006, 124, 124104 10.1063/1.2179420. 16599659

[ref42] Storchi L.; Belpassi L.; Tarantelli F.; Sgamellotti A.; Quiney H. M. An Efficient Parallel All-Electron Four-Component Dirac-Kohn-Sham Program Using a Distributed Matrix Approach. J. Chem. Theory Comput. 2010, 6, 384–394. 10.1021/ct900539m. 26617297

[ref43] Belpassi L.; De Santis M.; Quiney H. M.; Tarantelli F.; Storchi L. BERTHA: Implementation of a four-component Dirac-Kohn-Sham relativistic framework. J. Chem. Phys. 2020, 152, 164118 10.1063/5.0002831. 32357778

[ref44] Storchi L.; De Santis M.; Belpassi L. BERTHA and PyBERTHA: State of the Art for Full Four-Component Dirac-Kohn-Sham Calculations, Parallel Computing: Technology Trends, Proceedings of the International Conference on Parallel Computing, PARCO 2019, Prague, Czech Republic, September 10–13, 2019; pp 354–363.

[ref45] Lopata K.; Van Kuiken B. E.; Khalil M.; Govind N. Linear-Response and Real-Time Time-Dependent Density Functional Theory Studies of Core-Level Near-Edge X-Ray Absorption. J. Chem. Theory Comput. 2012, 8, 3284–3292. 10.1021/ct3005613. 26605735

[ref46] Ding F.; Van Kuiken B. E.; Eichinger B. E.; Li X. An efficient method for calculating dynamical hyperpolarizabilities using real-time time-dependent density functional theory. J. Chem. Phys. 2013, 138, 064104 10.1063/1.4790583. 23425458

[ref47] Cheng C.-L.; Evans J. S.; Van Voorhis T. Simulating molecular conductance using real-time density functional theory. Phys. Rev. B 2006, 74, 155112 10.1103/PhysRevB.74.155112.

[ref48] Isborn C. M.; Li X. Singlet-Triplet Transitions in Real-Time Time-Dependent Hartree-Fock/Density Functional Theory. J. Chem. Theory Comput. 2009, 5, 2415–2419. 10.1021/ct900264b. 26616622

[ref49] Goings J. J.; Li X. An atomic orbital based real-time time-dependent density functional theory for computing electronic circular dichroism band spectra. J. Chem. Phys. 2016, 144, 234102 10.1063/1.4953668. 27334149

[ref50] Peralta J. E.; Hod O.; Scuseria G. E. Magnetization Dynamics from Time-Dependent Noncollinear Spin Density Functional Theory Calculations. J. Chem. Theory Comput. 2015, 11, 3661–3668. 10.1021/acs.jctc.5b00494. 26574449

[ref51] Li X.; Tully J. C.; Schlegel H. B.; Frisch M. J. Ab initio Ehrenfest dynamics. J. Chem. Phys. 2005, 123, 084106 10.1063/1.2008258. 16164281

[ref52] Kolesov G.; Grånäs O.; Hoyt R.; Vinichenko D.; Kaxiras E. Real-Time TD-DFT with Classical Ion Dynamics: Methodology and Applications. J. Chem. Theory Comput. 2016, 12, 466–476. 10.1021/acs.jctc.5b00969. 26680129

[ref53] Kadek M.; Konecny L.; Gao B.; Repisky M.; Ruud K. X-ray absorption resonances near L2,3-edges from real-time propagation of the Dirac-Kohn-Sham density matrix. Phys. Chem. Chem. Phys. 2015, 17, 22566–22570. 10.1039/C5CP03712C. 26268195

[ref54] Konecny L.; Kadek M.; Komorovsky S.; Malkina O. L.; Ruud K.; Repisky M. Acceleration of Relativistic Electron Dynamics by Means of X2C Transformation: Application to the Calculation of Nonlinear Optical Properties. J. Chem. Theory Comput. 2016, 12, 5823–5833. 10.1021/acs.jctc.6b00740. 27792323

[ref55] Konecny L.; Kadek M.; Komorovsky S.; Ruud K.; Repisky M. Resolution-of-identity accelerated relativistic two- and four-component electron dynamics approach to chiroptical spectroscopies. J. Chem. Phys. 2018, 149, 204104 10.1063/1.5051032. 30501232

[ref56] Marques M. A. L.; López X.; Varsano D.; Castro A.; Rubio A. Time-Dependent Density-Functional Approach for Biological Chromophores: The Case of the Green Fluorescent Protein. Phys. Rev. Lett. 2003, 90, 258101 10.1103/PhysRevLett.90.258101. 12857170

[ref57] Liang W.; Chapman C. T.; Ding F.; Li X. Modeling Ultrafast Solvated Electronic Dynamics Using Time-Dependent Density Functional Theory and Polarizable Continuum Model. J. Phys. Chem. A 2012, 116, 1884–1890. 10.1021/jp2123899. 22277083

[ref58] Nguyen P. D.; Ding F.; Fischer S. A.; Liang W.; Li X. Solvated First-Principles Excited-State Charge-Transfer Dynamics with Time-Dependent Polarizable Continuum Model and Solvent Dielectric Relaxation. J. Phys. Chem. Lett. 2012, 3, 2898–2904. 10.1021/jz301042f.

[ref59] Pipolo S.; Corni S.; Cammi R. The cavity electromagnetic field within the polarizable continuum model of solvation: An application to the real-time time dependent density functional theory. Comput. Theor. Chem. 2014, 1040–1041, 112–119. 10.1016/j.comptc.2014.02.035.

[ref60] Corni S.; Pipolo S.; Cammi R. Equation of Motion for the Solvent Polarization Apparent Charges in the Polarizable Continuum Model: Application to Real-Time TDDFT. J. Phys. Chem. A 2015, 119, 5405–5416. 10.1021/jp5106828. 25485456

[ref61] Ding F.; Lingerfelt D. B.; Mennucci B.; Li X. Time-dependent non-equilibrium dielectric response in QM/continuum approaches. J. Chem. Phys. 2015, 142, 034120 10.1063/1.4906083. 25612702

[ref62] Donati G.; Wildman A.; Caprasecca S.; Lingerfelt D. B.; Lipparini F.; Mennucci B.; Li X. Coupling Real-Time Time-Dependent Density Functional Theory with Polarizable Force Field. J. Phys. Chem. Lett. 2017, 8, 5283–5289. 10.1021/acs.jpclett.7b02320. 28994290

[ref63] Wu X.; Teuler J.-M.; Cailliez F.; Clavaguéra C.; Salahub D. R.; de la Lande A. Simulating Electron Dynamics in Polarizable Environments. J. Chem. Theory Comput. 2017, 13, 3985–4002. 10.1021/acs.jctc.7b00251. 28738144

[ref64] Gil G.; Pipolo S.; Delgado A.; Rozzi C. A.; Corni S. Nonequilibrium Solvent Polarization Effects in Real-Time Electronic Dynamics of Solute Molecules Subject to Time-Dependent Electric Fields: A New Feature of the Polarizable Continuum Model. J. Chem. Theory Comput. 2019, 15, 2306–2319. 10.1021/acs.jctc.9b00010. 30860829 PMC6581418

[ref65] Koh K. J.; Nguyen-Beck T. S.; Parkhill J. Accelerating Realtime TDDFT with Block-Orthogonalized Manby-Miller Embedding Theory. J. Chem. Theory Comput. 2017, 13, 4173–4178. 10.1021/acs.jctc.7b00494. 28723221

[ref66] Lee S. J. R.; Welborn M.; Manby F. R.; Miller T. F. Projection-Based Wavefunction-in-DFT Embedding. Acc. Chem. Res. 2019, 52, 1359–1368. 10.1021/acs.accounts.8b00672. 30969117

[ref67] Gomes A. S. P.; Jacob C. R. Quantum-chemical embedding methods for treating local electronic excitations in complex chemical systems. Annu. Rep. Prog. Chem., Sect. C 2012, 108, 222 10.1039/c2pc90007f.

[ref68] Jacob C. R.; Neugebauer J. Subsystem density-functional theory. Wiley Interdiscip. Rev.: Comput. Mol. Sci. 2014, 4, 325–362. 10.1002/wcms.1175. PMC3920634 24563665

[ref69] Wesolowski T. A.; Shedge S.; Zhou X. Frozen-Density Embedding Strategy for Multilevel Simulations of Electronic Structure. Chem. Rev. 2015, 115, 5891–5928. 10.1021/cr500502v. 25923542

[ref70] Krishtal A.; Ceresoli D.; Pavanello M. Subsystem real-time time dependent density functional theory. J. Chem. Phys. 2015, 142, 154116 10.1063/1.4918276. 25903875

[ref71] Wesolowski T. A.; Warshel A. Frozen density functional approach for ab initio calculations of solvated molecules. J. Phys. Chem. A. 1993, 97, 8050–8053. 10.1021/j100132a040.

[ref72] Senatore G.; Subbaswamy K. R. Density dependence of the dielectric constant of rare-gas crystals. Phys. Rev. B 1986, 34, 5754–5757. 10.1103/PhysRevB.34.5754. 9940414

[ref73] Cortona P. Direct determination of self-consistent total energies and charge densities of solids: A study of the cohesive properties of the alkali halides. Phys. Rev. B 1992, 46, 2008–2014. 10.1103/PhysRevB.46.2008. 10003874

[ref74] Iannuzzi M.; Kirchner B.; Hutter J. Density functional embedding for molecular systems. Chem. Phys. Lett. 2006, 421, 16–20. 10.1016/j.cplett.2005.08.155.

[ref75] Jacob C. R.; Neugebauer J.; Visscher L. A flexible implementation of frozen-density embedding for use in multilevel simulations. J. Comput. Chem. 2008, 29, 1011–1018. 10.1002/jcc.20861. 17987602

[ref76] Casida M. E.; Wesolowski T. A. Generalization of the Kohn-Sham equations with constrained electron density formalism and its time-dependent response theory formulation. Int. J. Quantum Chem. 2004, 96, 577–588. 10.1002/qua.10744.

[ref77] Neugebauer J. Couplings between electronic transitions in a subsystem formulation of time-dependent density functional theory. J. Chem. Phys. 2007, 126, 134116 10.1063/1.2713754. 17430025

[ref78] Neugebauer J. On the calculation of general response properties in subsystem density functional theory. J. Chem. Phys. 2009, 131, 084104 10.1063/1.3212883. 19725605

[ref79] Tölle J.; Böckers M.; Neugebauer J. Exact subsystem time-dependent density-functional theory. J. Chem. Phys. 2019, 150, 181101 10.1063/1.5097124. 31091916

[ref80] Tölle J.; Böckers M.; Niemeyer N.; Neugebauer J. Inter-subsystem charge-transfer excitations in exact subsystem time-dependent density-functional theory. J. Chem. Phys. 2019, 151, 174109 10.1063/1.5121908. 31703509

[ref81] Fux S.; Jacob C. R.; Neugebauer J.; Visscher L.; Reiher M. Accurate frozen-density embedding potentials as a first step towards a subsystem description of covalent bonds. J. Chem. Phys. 2010, 132, 164101 10.1063/1.3376251. 20441252

[ref82] Goodpaster J. D.; Ananth N.; Manby F. R.; Miller T. F. Exact nonadditive kinetic potentials for embedded density functional theory. J. Chem. Phys. 2010, 133, 084103 10.1063/1.3474575. 20815556

[ref83] Goodpaster J. D.; Barnes T. A.; Miller T. F. Embedded density functional theory for covalently bonded and strongly interacting subsystems. J. Chem. Phys. 2011, 134, 164108 10.1063/1.3582913. 21528951

[ref84] Huang C.; Pavone M.; Carter E. A. Quantum mechanical embedding theory based on a unique embedding potential. J. Chem. Phys. 2011, 134, 154110 10.1063/1.3577516. 21513378

[ref85] Nafziger J.; Wu Q.; Wasserman A. Molecular binding energies from partition density functional theory. J. Chem. Phys. 2011, 135, 234101 10.1063/1.3667198. 22191858

[ref86] Jacob C. R.; Beyhan S. M.; Bulo R. E.; Gomes A. S. P.; Götz A. W.; Kiewisch K.; Sikkema J.; Visscher L. PyADF – A scripting framework for multiscale quantum chemistry. J. Comput. Chem. 2011, 32, 2328–2338. 10.1002/jcc.21810. 21541961

[ref87] Ekström U. XCFun: Exchange-Correlation functionals with arbitrary order derivatives, 2019. https://github.com/dftlibs/xcfun.

[ref88] Thomas L. H. The calculation of atomic fields. Math. Proc. Cambridge Philos. Soc. 1927, 23, 542–548. 10.1017/S0305004100011683.

[ref89] Lembarki A.; Chermette H. Obtaining a gradient-corrected kinetic-energy functional from the Perdew-Wang exchange functional. Phys. Rev. A 1994, 50, 5328–5331. 10.1103/PhysRevA.50.5328. 9911536

[ref90] Mi W.; Pavanello M. Nonlocal Subsystem Density Functional Theory.. J. Phys. Chem. Lett. 2020, 11, 272–279. 10.1021/acs.jpclett.9b03281. 31820994

[ref91] Gomes A. S. P.; Jacob C. R.; Visscher L. Calculation of local excitations in large systems by embedding wave-function theory in density-functional theory. Phys. Chem. Chem. Phys. 2008, 10, 5353–5362. 10.1039/b805739g. 18766231

[ref92] Bouchafra Y.; Shee A.; Réal F.; Vallet V.; Gomes A. S. P. Predictive simulations of ionization energies of solvated halide ions with relativistic embedded Equation of Motion Coupled Cluster Theory. Phys. Rev. Lett. 2018, 121, 266001 10.1103/PhysRevLett.121.266001. 30636145

[ref93] Halbert L.; Olejniczak M.; Vallet V.; Gomes A. S. P. Investigating solvent effects on the magnetic properties of molybdate ions (MoO_4_^2–^) with relativistic embedding. Int. J. Quantum Chem. 2019, e26207 10.1002/qua.26207.

[ref94] Höfener S.; Severo Pereira Gomes A.; Visscher L. Molecular properties via a subsystem density functional theory formulation: A common framework for electronic embedding. J. Chem. Phys. 2012, 136, 044104 10.1063/1.3675845. 22299858

[ref95] Olejniczak M.; Bast R.; Gomes A. S. P. On the calculation of second-order magnetic properties using subsystem approaches in a relativistic framework. Phys. Chem. Chem. Phys. 2017, 19, 8400–8415. 10.1039/C6CP08561J. 28282090

[ref96] Neugebauer J.; Jacob C. R.; Wesolowski T. A.; Baerends E. J. An Explicit Quantum Chemical Method for Modeling Large Solvation Shells Applied to Aminocoumarin C151. J. Phys. Chem. A 2005, 109, 7805–7814. 10.1021/jp0528764. 16834158

[ref97] Bulo R. E.; Jacob C. R.; Visscher L. NMR Solvent Shifts of Acetonitrile from Frozen Density Embedding Calculations. J. Phys. Chem. A 2008, 112, 2640–2647. 10.1021/jp710609m. 18302351

[ref98] Castro A.; Marques M. A. L.; Rubio A. Propagators for the time-dependent Kohni-Sham equations. J. Chem. Phys. 2004, 121, 3425–3433. 10.1063/1.1774980. 15303905

[ref99] Meng S.; Kaxiras E. Real-time, local basis-set implementation of time-dependent density functional theory for excited state dynamics simulations. J. Chem. Phys. 2008, 129, 054110 10.1063/1.2960628. 18698891

[ref100] Press W. H.; Teukolsky S. A.; Vetterling W. T.; Flannery B. P. Numerical Recipes 3rd Edition: The Art of Scientific Computing; Cambridge University Press, 2007.

[ref101] Magnus W. On the exponential solution of differential equations for a linear operator. Commun. Pure Appl. Math. 1954, 7, 649–673. 10.1002/cpa.3160070404.

[ref102] Casas F.; Iserles A. Explicit Magnus expansions for nonlinear equations. J. Phys. A: Math. Gen. 2006, 39, 5445–5461. 10.1088/0305-4470/39/19/S07.

[ref103] Zhu Y.; Herbert J. M. Self-consistent predictor/corrector algorithms for stable and efficient integration of the time-dependent Kohn-Sham equation. J. Chem. Phys. 2018, 148, 044117 10.1063/1.5004675. 29390834

[ref104] Bandrauk A. D.; Chelkowski S.; Diestler D. J.; Manz J.; Yuan K.-J. Quantum simulation of high-order harmonic spectra of the hydrogen atom. Phys. Rev. A 2009, 79, 023403 10.1103/PhysRevA.79.023403.

[ref105] Jacob C. R.; Beyhan S. M.; Bulo R. E.; Gomes A. S. P.; Goetz A.; Handzlik M.; Kiewisch K.; Klammler M.; Sikkema J.; Visscher L. PyADF – A Scripting Framework for Multiscale Quantum Chemistry: Version 0.96, 2020. https://github.com/chjacob-tubs/pyadf-releases, DOI: 10.5281/zenodo.3834283. 21541961

[ref106] Gomes A. S. P.; Jacob C. R. PyEmbed – A Frozen-Density Embedding Module for PyADF, 2020. 10.5281/zenodo.3834283.

[ref107] Schmitt-Monreal D.; Jacob C. R. Frozen-density embedding-based many-body expansions. Int. J. Quantum Chem. 2020, e26228 10.1002/qua.26228.

[ref108] De Santis M. git URL: https://github.com/lstorchi/pybertha/tree/master/psi4embedrt within the PyBertha project: https://github.com/lstorchi/pybertha written by Storchi L.; De Santis M.; Belpassi L..

[ref109] De Santis M. Numerical data and post-processing material, 2020. 10.5281/zenodo.3885610.

[ref110] Parrish R. M.; Burns L. A.; Smith D. G. A.; Simmonett A. C.; DePrince A. E.; Hohenstein E. G.; Bozkaya U.; Sokolov A. Y.; Di Remigio R.; Richard R. M.; Gonthier J. F.; James A. M.; McAlexander H. R.; Kumar A.; Saitow M.; Wang X.; Pritchard B. P.; Verma P.; Schaefer H. F.; Patkowski K.; King R. A.; Valeev E. F.; Evangelista F. A.; Turney J. M.; Crawford T. D.; Sherrill C. D. Psi4 1.1: An Open-Source Electronic Structure Program Emphasizing Automation, Advanced Libraries, and Interoperability. J. Chem. Theory Comput. 2017, 13, 3185–3197. 10.1021/acs.jctc.7b00174. 28489372 PMC7495355

[ref111] Storchi L. Python 3 port of PyADF v0.96, 2020. 10.5281/zenodo.3834286.

[ref112] Storchi L. Python 3 port of XcFun a486a3f148, 2020. https://github.com/lstorchi/xcfun.

[ref113] Baerends E. J.; Ziegler T.; Atkins A. J.; Autschbach J.; Bashford D.; Baseggio O.; Bérces A.; Bickelhaupt F. M.; Bo C.; Boerritger P. M.; Cavallo L.; Daul C.; Chong D. P.; Chulhai D. V.; Deng L.; Dickson R. M.; Dieterich J. M.; Ellis D. E.; van Faassen M.; Ghysels A.; Giammona A.; van Gisbergen S. J. A.; Goez A.; Götz A. W.; Gusarov S.; Harris F. E.; van den Hoek P.; Hu Z.; Jacob C. R.; Jacobsen H.; Jensen L.; Joubert L.; Kaminski J. W.; van Kessel G.; König C.; Kootstra F.; Kovalenko A.; Krykunov M.; van Lenthe E.; McCormack D. A.; Michalak A.; Mitoraj M.; Morton S. M.; Neugebauer J.; Nicu V. P.; Noodleman L.; Osinga V. P.; Patchkovskii S.; Pavanello M.; Peeples C. A.; Philipsen P. H. T.; Post D.; Pye C. C.; Ramanantoanina H.; Ramos P.; Ravenek W.; Rodríguez J. I.; Ros P.; Rüger R.; Schipper P. R. T.; Schlüns D.; van Schoot H.; Schreckenbach G.; Seldenthuis J. S.; Seth M.; Snijders J. G.; Solà M.; M S.; Swart M.; Swerhone D.; te Velde G.; Tognetti V.; Vernooijs P.; Versluis L.; Visscher L.; Visser O.; Wang F.; Wesolowski T. A.; van Wezenbeek E. M.; Wiesenekker G.; Wolff S. K.; Woo T. K.; Yakovlev A. L. ADF2017, SCM, Theoretical Chemistry; Vrije Universiteit, Amsterdam, The Netherlands. https://www.scm.com.

[ref114] Dułak M.; Kamiński J. W.; Wesolowski T. A. Linearized orbital-free embedding potential in self-consistent calculations. Int. J. Quantum Chem. 2009, 109, 1886–1897. 10.1002/qua.22011.

[ref115] Wesolowski T. A. Hydrogen-Bonding-Induced Shifts of the Excitation Energies in Nucleic Acid Bases: An Interplay between Electrostatic and Electron Density Overlap Effects. J. Am. Chem. Soc. 2004, 126, 11444–11445. 10.1021/ja048846g. 15366883

[ref116] Te Velde Gt.; Bickelhaupt F. M.; Baerends E. J.; Fonseca Guerra C.; van Gisbergen S. J.; Snijders J. G.; Ziegler T. Chemistry with ADF. J. Comput. Chem. 2001, 22, 931–967. 10.1002/jcc.1056.

[ref117] Bruner A.; LaMaster D.; Lopata K. Accelerated broadband spectra using transition dipole decomposition and Padé approximants. J. Chem. Theory Comput. 2016, 12, 3741–3750. 10.1021/acs.jctc.6b00511. 27359347

[ref118] Dunning T. H. Gaussian basis sets for use in correlated molecular calculations. I. The atoms boron through neon and hydrogen. J. Chem. Phys. 1989, 90, 1007–1023. 10.1063/1.456153.

[ref119] Kendall R. A.; Dunning T. H.; Harrison R. J. Electron affinities of the first row atoms revisited. Systematic basis sets and wave functions. J. Chem. Phys. 1992, 96, 6796–6806. 10.1063/1.462569.

[ref120] Becke A. D. Density-functional exchange-energy approximation with correct asymptotic behavior. Phys. Rev. A 1988, 38, 3098–3100. 10.1103/PhysRevA.38.3098. 9900728

[ref121] Lee C.; Yang W.; Parr R. G. Development of the Colle-Salvetti correlation-energy formula into a functional of the electron density. Phys. Rev. B 1988, 37, 785–789. 10.1103/PhysRevB.37.785. 9944570

[ref122] Vosko S. H.; Wilk L.; Nusair M. Accurate spin-dependent electron liquid correlation energies for local spin density calculations: a critical analysis. Can. J. Phys. 1980, 58, 1200–1211. 10.1139/p80-159.

[ref123] Slater J. C. A Simplification of the Hartree-Fock Method. Phys. Rev. 1951, 81, 385–390. 10.1103/PhysRev.81.385.

[ref124] Jacob C. R.; Neugebauer J.; Jensen L.; Visscher L. Comparison of frozen-density embedding and discrete reaction field solvent models for molecular properties. Phys. Chem. Chem. Phys. 2006, 8, 2349–2359. 10.1039/b601997h. 16710483

[ref125] Höfener S.; Gomes A. S. P.; Visscher L. Solvatochromic shifts from coupled-cluster theory embedded in density functional theory. J. Chem. Phys. 2013, 139, 104106 10.1063/1.4820488. 24050327

[ref126] Pram S. K.; Genova A.; Pavanello M. Cooperation and Environment Characterize the Low-Lying Optical Spectrum of Liquid Water. J. Phys. Chem. Lett. 2017, 8, 5077–5083. 10.1021/acs.jpclett.7b02212. 28968128

[ref127] Luppi E.; Head-Gordon M. Computation of high-harmonic generation spectra of H2 And N2 in intense laser pulses using quantum chemistry methods and time-dependent density functional theory. Mol. Phys. 2012, 110, 909–923. 10.1080/00268976.2012.675448.

[ref128] Lewenstein M.; Balcou P.; Ivanov M. Y.; L’Huillier A.; Corkum P. B. Theory of high-harmonic generation by low-frequency laser fields. Phys. Rev. A 1994, 49, 2117–2132. 10.1103/PhysRevA.49.2117. 9910464

[ref129] Barth S.; Oncak M.; Ulrich V.; Mucke M.; Lischke T.; Slavicek P.; Hergenhahn U. Valence Ionization of Water Clusters: From Isolated Molecules to Bulk. J. Phys. Chem. A 2009, 113, 13519–13527. 10.1021/jp906113e. 19856943

[ref130] Klahr K.; Schlüns D.; Neugebauer J. Geometry Optimizations in a Subsystem Density Functional Theory Formalism: A Benchmark Study. J. Chem. Theory Comput. 2018, 14, 5631–5644. 10.1021/acs.jctc.8b00475. 30272968

