# Supplementary material for: Environmental Effects with Frozen-Density Embedding in Real-Time Time-Dependent Density Functional Theory Using Localized Basis Functions
Source: J Chem Theory Comput. 2020 Jul 27;16(9):5695–711. doi: 10.1021/acs.jctc.0c00603 (PMC8009524; doi:10.1021/acs.jctc.0c00603)
Supplement: Supplementary file 1 — ct0c00603_si_001.pdf [file ct0c00603_si_001.pdf]

Supporting Information: Environmental effects with Frozen Density Embedding in Real-Time Time-Dependent Density Functional Theory using localized basis functions.

Matteo De Santis,[*,†,‡] Leonardo Belpassi,[‡] Christoph R. Jacob,[¶] André Severo Pereira Gomes,[§] Francesco Tarantelli,[†] Lucas Visscher,[‖] and Loriano Storchi[⊥,‡]

†*Dipartimento di Chimica, Biologia e Biotecnologie, Università degli Studi di Perugia, Via Elce di Sotto 8, 06123 Perugia, Italy*

‡*Istituto di Scienze e Tecnologie Chimiche (SCITEC), Consiglio Nazionale delle Ricerche c/o Dipartimento di Chimica, Biologia e Biotecnologie, Università degli Studi di Perugia, Via Elce di Sotto 8, 06123 Perugia, Italy*

¶*Institute of Physical and Theoretical Chemistry, Technische Universität Braunschweig, Gaußstr. 17, 38106 Braunschweig, Germany*

§*Univ. Lille, CNRS, UMR 8523-PhLAM-Physique des Lasers Atomes et Molécules, F-59000 Lille, France*

‖*Theoretical Chemistry, Faculty of Science, Vrije Universiteit Amsterdam, De Boelelaan 1083, NL-1081HV Amsterdam, Netherlands*

⊥*Dipartimento di Farmacia, Università degli Studi 'G. D'Annunzio', Via dei Vestini 31, 66100 Chieti, Italy*

E-mail: matteo.des89@gmail.com

Contents

1 Real-time propagation pseudocode **3**

2 Psi4-RT & NWChem-LR vs ADF-LR **4**

3 Basis set convergence **5**

1 Real-time propagation pseudocode

In the following we are reporting the pseudocode we used to solve the time dependent equation of motion using the second-order midpoint Magnus propagator as illustrated in Section 2.2.

Algorithm 1 Predictor-corrector ME algorithm

Inputs : $\mathbf{D}(t_i), \mathbf{F}(t_{i-1/2})$
set : $\mathbf{F}_{-1/2} \leftarrow \mathbf{F}_0$
for $i = 0$ to $Niter$ **do**
    $evaluate\, \mathbf{F}(t_i)\, from\, \mathbf{D}(t_i)$
    $\mathbf{F}(t_i) = \mathbf{F}(t_i)\, (+\mathbf{V}_{emb}[D(t_i), D_{env}(0), v_{env}^{coul,nuc}])$
    $\mathbf{F}(t_{i+1/2}) \leftarrow 2\mathbf{F}(t_i) - \mathbf{F}(t_{i-1/2})$
    $counter = 1$
    loop
        $\mathbf{U}_i = \mathbf{U}(t_{i+1/2}, t_i) \leftarrow \mathbf{F}(t_{i+1/2})$
        $\mathbf{D}(t_{i+1}) \leftarrow \mathbf{U}_i \mathbf{D}(t_i) \mathbf{U}_i^\dagger$
        $evaluate\, \mathbf{F}(t_{i+1}) from\, \mathbf{D}(t_{i+1})$
        $\mathbf{F}(t_{i+1/2}) \leftarrow 0.5\mathbf{F}(t_i) + 0.5\mathbf{F}(t_{i+1})\, (+\mathbf{V}_{emb}[D(t_i), D_{env}(0), v_{env}^{coul,nuc}])$
        if $counter > 1$ **then**
            $\delta\mathbf{D}(t_{i+1}) \leftarrow \mathbf{D}(t_{i+1}) - \mathbf{D}_{test}$
            if $||\delta\mathbf{D}(t_{i+1})||_F < eps$ **then**
                exit the loop
            end if
        end if
        $\mathbf{D}_{test} = \mathbf{D}(t_{i+1})$
        $counter+ = 1$
    end loop
end for
Output : $\mathbf{D}(t_{i+1}), \mathbf{F}(t_{i+1/2})$

2 Psi4-RT & NWChem-LR vs ADF-LR

Table S1: Excitation energies for isolated water molecule. We compare the data from our reference implementation of RT-TDDFT (Psi4-rt) with those obtained using TDDFT based on linear response implemented in NWChem and ADF. In the calculation using Psi4-rt and NWChem aug-cc-pVDZ basis set was used, wheras in the ADF calculation AUG-DZP basis set was used.

	Excitation energy (e.V)		
	NWChem-LR	Psi4-rt	ADF-LR
Root 1	6.214	6.215	6.161
Root 2	7.513	7.512	7.454
Root 3	8.363	8.363	8.309
Root 4	9.536	9.536	8.803
Root 5	9.644	9.644	8.945
Root 6	10.405	10.405	9.353
Root 7	10.478	10.476	9.588
Root 8	10.699	10.699	10.071
Root 9	11.524	11.524	10.700
Root 10	11.994	11.993	10.994

3 Basis set convergence

Table S2: Excitation energies for isolated water molecule calculated using our reference implementation of RT-TDDFT (Psi4-rt) and linear response implemented in ADF are reported. The labels (D,T,Q) correspond to data obtained using the Gaussian-type basis sets aug-cc-pVXZ (X = D,T,Q) and Slater-type basis sets AUG-X′ (X′ = DZP,TZ2P,QZ4P) which are used in the Psi4-rt and ADF-LR codes, respectively.

	Excitation energy (e.V)					
	Psi4-rt			ADF-LR		
	D	T	Q	D	T	Q
Root 1	6.215	6.227	6.224	6.161	6.189	6.287
Root 2	7.512	7.466	7.440	7.454	7.465	7.884
Root 3	8.363	8.352	8.344	8.309	8.288	8.427
Root 4	9.536	8.953	8.651	8.803	8.482	8.628
Root 5	9.644	9.572	9.306	8.945	8.845	10.022
Root 6	10.405	9.696	9.371	9.353	8.921	10.870
Root 7	10.476	9.817	9.523	9.588	9.555	11.104
Root 8	10.699	9.888	9.545	10.071	9.559	11.585
Root 9	11.524	10.874	10.348	10.700	10.077	12.250
Root 10	11.993	11.277	10.673	10.994	10.796	12.522

Table S3: Excitation energies (in eV) corresponding to the first ten low-lying transitions of both the isolated and embedded water molecule are reported. In the embedded water molecule an ammonia is used as environment. Data have been obtained using our new Psi4-rt-PyEmbed implementation with aug-cc-pVDZ basis set and with the reference ADF-LR-FDE implementation in combination with the AUG-DZP basis set. The shift $\Delta(E_{iso.}-E_{emb})$ in the transition energies due to the embedding environment is also reported.

Excitation energy (e.V)						
	Psi4-RT-PyEmbed			ADF-LR-FDE		
	isolated	emb.	Δ	isolated	emb.	Δ
Root 1	6.215	5.817	0.398	6.161	5.687	0.474
Root 2	7.512	6.694	0.818	7.454	6.578	0.876
Root 3	8.363	7.892	0.470	8.309	7.782	0.527
Root 4	9.536	8.768	0.768	8.803	8.336	0.467
Root 5	9.644	9.186	0.458	8.945	8.422	0.523
Root 6	10.405	10.008	0.396	9.353	8.656	0.697
Root 7	10.476	10.048	0.428	9.588	8.855	0.733
Root 8	10.699	10.615	0.084	10.071	9.866	0.205
Root 9	11.524	11.358	0.166	10.700	10.433	0.267
Root 10	11.993	11.589	0.405	10.994	10.446	0.548

Table S4: Excitation energies (in eV) corresponding to the first ten low-lying transitions of both the isolated and embedded water molecule are reported. In the embedded water molecule an ammonia is used as environment. Data have been obtained using our new Psi4-rt-PyEmbed implementation with aug-cc-pVTZ basis set and with the reference ADF-LR-FDE implementation in combination with the AUG-TZ2P basis set. The shift $\Delta(E_{iso.}-E_{emb})$ in the transition energies due to the embedding environment is also reported.

Excitation energy (e.V)						
	Psi4-RT-PyEmbed			ADF-LR-FDE		
	isolated	emb.	Δ	isolated	emb.	Δ
Root 1	6.227	5.796	0.430	6.189	5.689	0.500
Root 2	7.466	6.573	0.893	7.465	6.559	0.905
Root 3	8.352	7.848	0.503	8.288	7.734	0.554
Root 4	8.953	8.560	0.393	8.482	7.969	0.513
Root 5	9.572	8.625	0.948	8.845	8.318	0.527
Root 6	9.696	9.213	0.483	8.921	8.348	0.573
Root 7	9.817	9.343	0.474	9.555	8.614	0.941
Root 8	9.888	9.726	0.162	9.559	8.832	0.727
Root 9	10.874	10.535	0.339	10.077	10.034	0.043
Root 10	11.277	10.850	0.427	10.796	10.164	0.632
